# Genetic dynamics in untreated CLL patients with either stable or progressive disease: a longitudinal study

**DOI:** 10.1186/s13045-019-0802-x

**Published:** 2019-11-19

**Authors:** Alice Ramassone, Andrea D’Argenio, Angelo Veronese, Alessio Basti, Shimaa Hassan AbdelAziz Soliman, Stefano Volinia, Cristian Bassi, Sara Pagotto, Manuela Ferracin, Laura Lupini, Elena Saccenti, Veronica Balatti, Felice Pepe, Laura Z. Rassenti, Idanna Innocenti, Francesco Autore, Laura Marzetti, Renato Mariani-Costantini, Thomas J. Kipps, Massimo Negrini, Luca Laurenti, Rosa Visone

**Affiliations:** 1 Unit of General Pathology, Center for Advanced Studies and Technology (CAST), University G. d’Annunzio Chieti-Pescara, Chieti, Italy; 2 Department of Medicine and Aging Sciences, University G. d’Annunzio Chieti-Pescara, Chieti, Italy; 3 Department of Neuroscience, Imaging and Clinical Sciences, University G. d’Annunzio Chieti-Pescara, Chieti, Italy; 4 Institute for Advanced Biomedical Technologies (ITAB), University G. d’Annunzio Chieti-Pescara, Chieti, Italy; 5 Department of Morphology, Surgery and Experimental Medicine, University of Ferrara, Ferrara, Italy; 6 Department of Medical, Oral and Biotechnological Sciences, University G. d’Annunzio Chieti-Pescara, Chieti, Italy; 7 Department of Experimental, Diagnostic and Specialty Medicine, University of Bologna, Bologna, Italy; 8 Department of Cancer Biology and Genetics and Comprehensive Cancer Center at the Wexner Medical Center, The Ohio State University, Columbus, OH USA; 9 Department of Medicine, Moores Cancer Center, University of California at San Diego, La Jolla, CA USA; 10 Chronic Lymphocytic Leukemia Research Consortium, San Diego, CA USA; 11 Fondazione Policlinico Universitario A Gemelli IRCCS, Rome, Italy

**Keywords:** Chronic lymphocytic leukemia, Copy number variation, Nucleotide variation, Clonal evolution

## Abstract

Clonal evolution of chronic lymphocytic leukemia (CLL) often follows chemotherapy and is associated with adverse outcome, but also occurs in untreated patients, in which case its predictive role is debated. We investigated whether the selection and expansion of CLL clone(s) precede an aggressive disease shift. We found that clonal evolution occurs in all CLL patients, irrespective of the clinical outcome, but is faster during disease progression. In particular, changes in the frequency of nucleotide variants (NVs) in specific CLL-related genes may represent an indicator of poor clinical outcome.

To the Editor

In chronic lymphocytic leukemia (CLL), the clonal expansion acquired relevance with the NGS era, which allowed its use for clinical monitoring. Research was mainly performed on large CLL cohorts sampled before and after therapy [1] and only a few studies investigated clonal evolution longitudinally in stable versus progressive untreated patients [2–4]. The key results indicate expansion of specific clones upon therapy and heterogeneity of mutated genes among patients, but the extent to which the genetic dynamics differs between stable and progressive untreated CLLs is still controversial.

To address this point, we used a CLL cohort including untreated sequential samples from patients with either progressive (P-CLL) or stable (S-CLL) disease. Patients’ features are in Additional file 1: Table S1. At each time point, the diagnosis of stable or progressive CLL was established by the clinicians according to the criteria defined during the International Workshop on Chronic Lymphocytic Leukemia [5]. Using genome-wide copy number variation (CNV) analysis, we investigated copy number fluctuations in 11 stable CLLs (S-CLLs) and 15 progressive CLLs (P-CLLs). Data were processed using the Rawcopy package [6], and paired segments were defined for each patient (Additional file 2: Figure S1). Since the percentage of CLL cells (*f*) in PBMCs was not always known, analyses were performed varying *f* from 1 to 100%. To define aberrant loci, we used two sets of thresholds on log ratio (LogR) value, depending on *f* and on copy number (*k*) in CLL cells (Additional file 2: Figure S2). We did not find significant differences in percentages of aberrant loci between S-CLLs and P-CLLs (Fig. 1a), but the rate of change (or slope), reflecting the rate of aberrant clones evolving over time, was significantly higher in P-CLLs (*p* ≤ 0.05, Mann-Whitney *U* test) (Fig. 1b). Thus, S-CLLs and P-CLLs seemed to have the same probability of acquiring or losing clones, but this phenomenon was faster in P-CLLs. The results were validated on 6 S-CLLs and 5 P-CLLs with known percentage of CLL cells in PBMCs (Additional file 2: Figure S3-S4), suggesting that tracking copy number changes does not mandatorily require knowledge of cancer cell percentage.

**Fig. 1:**
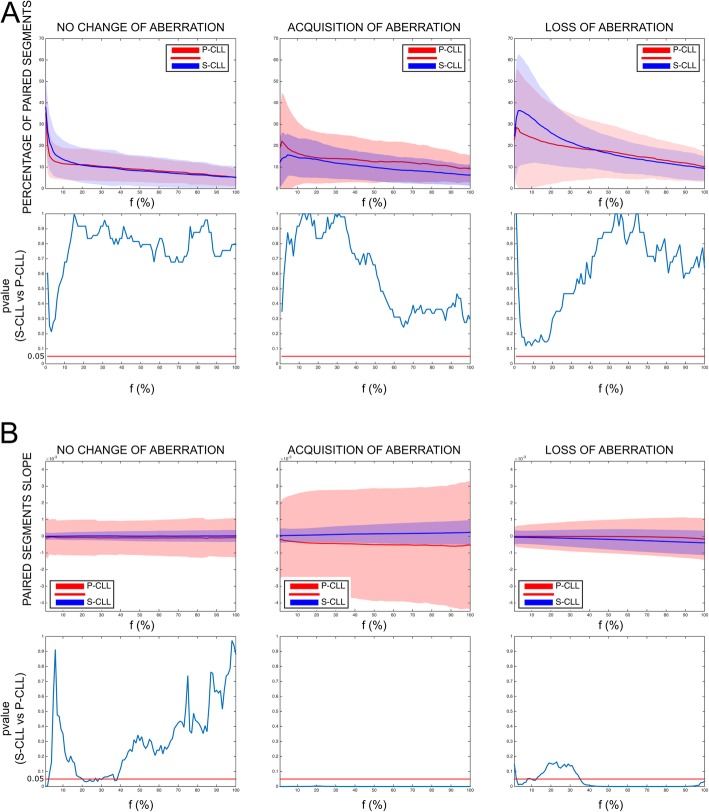
Longitudinal analysis of copy number aberrations in stable and progressive CLL patients. Genome-Wide Human SNP Arrays 6.0 (Affymetrix) was used to genotype patient DNAs at the two sequential sampling points, first time point (FTP), and last time point (LTP). Data were processed using the Rawcopy package, and paired segments (PSs) were identified between FTP and LTP of each patient (see Methods in Additional file 2). Aberrant loci were identified by varying the percentage of cancer cells (*f)* using two sets of threshold on log ratio (LogR), one for DNA amplification (LogR_A_) and one for DNA deletion (LogR_D_). **a** The percentage of PSs and **b** their slopes (*∆*LogR_LTP, FTP_/*∆t*_LTP, FTP_) are shown as mean (solid line) and standard deviation (shades) based on no change, acquisition, or loss of aberration as a function of *f***.** No change of aberration: the considered locus was aberrant both in FTP and in LTP; acquisition of aberrations: the considered locus was aberrant only in LTP; loss of aberrations: the considered locus was aberrant only in FTP. Red colors indicate the P-CLLs; blue the S-CLLs. *p* values’ graphs (lower panel) report the Mann-Whitney *U* test for each *f*, significance was defined as *p* < 0.050

To identify genetic events associated with faster clonal expansion, we characterized the CLL-specific genetic features of our cohort. Analyses by qPCR of three chromosomal abnormalities of prognostic value, del (11q), tri (12), and del (17p) [7], did not reveal significant differences between S-CLLs and P-CLLs (Additional file 2: Figure S5). Subsequently, we characterized 11 S-CLLs and 17 P-CLLs for point mutations or indels in regions of 27 genes reported as mutated in CLL (Additional file 3: Table S2). We did not register any significant difference between S-CLLs and P-CLLs with regard to frequency and number of nucleotide variants (NVs) (data not shown). Next, we focused only on NVs with variant allele frequencies (VAF) changing more than 20% between longitudinal samples (dynamic NV: dNVs, synonymous or non-synonymous). We detected on average 1.18 and 3.35 dNVs per sample in S-CLLs and P-CLLs, respectively (Additional file 4: Table S3). P-CLLs showed higher gains/increases of dNVs (*p* = 0.0008, Fisher’s test) (Fig. 2a). Patients with dNV > 1 had shorter treatment-free survival (TFS), considering as starting point the date at first sampling (*p* = 0.0029) or at diagnosis (*p* = 0.0004, log rank test) (Fig. 2b and Additional file 2: Figure S6). A dNV > 1 was also associated with poor prognostic factors, including unmutated *IGVH* and trisomy 12 (*p* = 0.0461 and *p* = 0.0407, respectively, Fisher’s test) (Additional file 5: Table S4). Patients with unmutated *IGVH* showed shorter TFS, supporting the reliability of our cohort (Fig. 2b). Finally, we found that in P-CLLs the average of dNV frequencies was higher in the first sample (*p* = 0.0074, Mann-Whitney *U* test), where it was not associated with *IGVH* mutational status (Fig. 2c). These findings suggest that dNVs could have an exploitable clinical relevance. However, since dNVs include synonymous/non-synonymous mutations and NVs in non-coding regions, we cannot speculate on the molecular role of the targeted genes most frequently mutated, such as *ITPKB* and *NOTCH1* (Fig. 2a). Indeed, these dNVs were only used here to track genetic evolution.

**Fig. 2:**
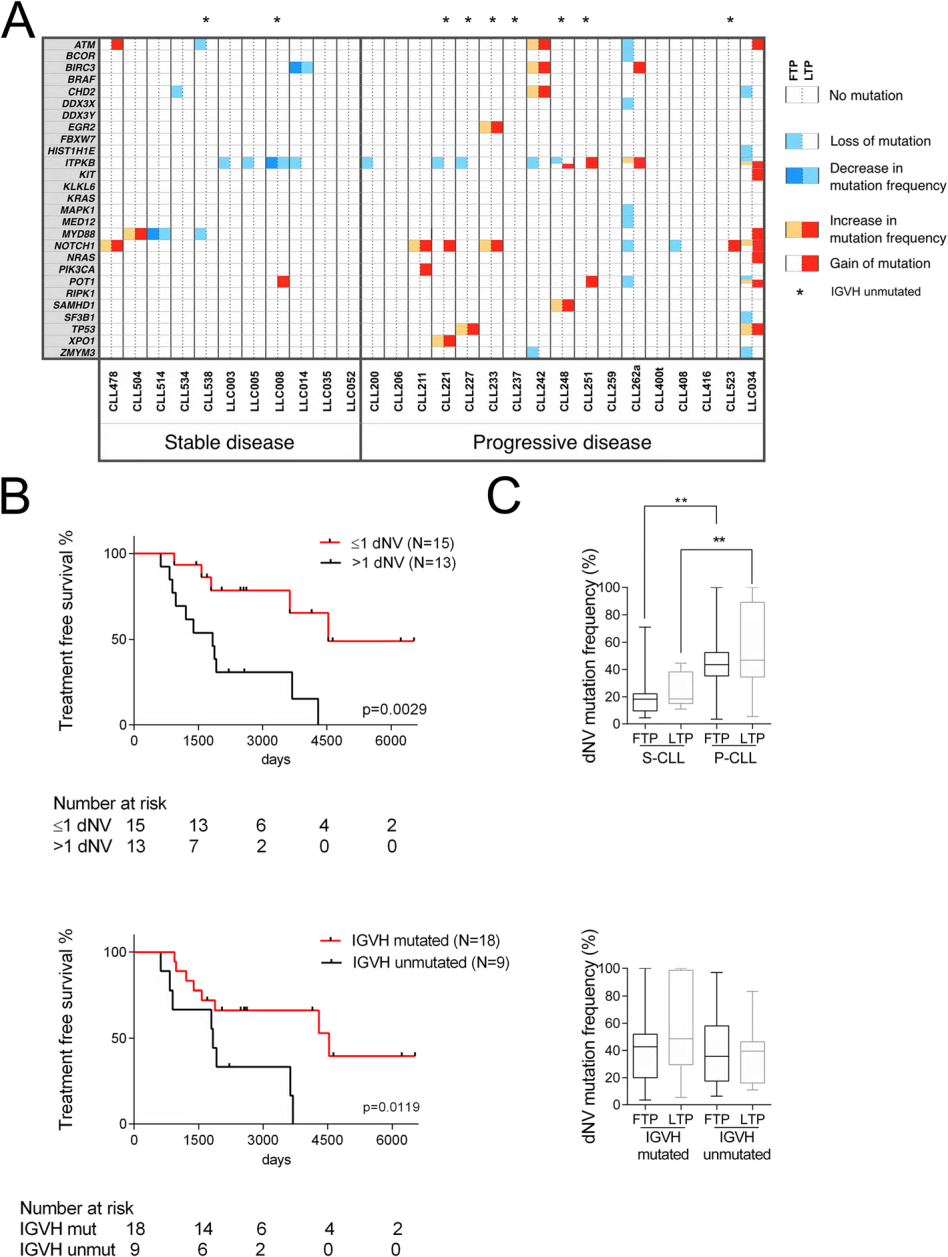
Mutational status of CLL samples. **a** Next-generation sequencing of 27 CLL-associated genes in 11 and 17 patients with stable and progressive disease, respectively. Sequence variants were identified using Torrent Suite 3.4 and Variant Caller plugin 3.4.4. NVs with a coverage < 100 were not considered; NVs with a mutation frequency < 5% were not considered; NVs residing in homopolymer DNA sequences (≥ 4 nucleotides) were also not considered. Each square report either gain or loss of mutation or change in mutation frequency (> 20%) between FTP (left side) and LTP (right side); the mutation frequency of dynamic nucleotide variants (dNVs) was reported in Additional file 4: Table S3. Fisher’s exact tests were used to compare groups. Statistical tests were two-sided, and significance was defined as *p* < 0.050. **b** Kaplan-Meier curve of treatment-free survival in CLL patients dichotomized based on the number of NVs that change more than 20% between FTP and LTP (dNVs) (upper panel) and on the mutational status of the poor prognostic factor *IGVH* (lower panel). The median of dNVs changed across all the samples was used as cut-off. Time to treatment was calculated from the first sampling; the last follow-up was considered for patients, which did not undergo treatment. The Log-rank test was used to test for significance. **c** Mutation frequency of dNVs in stable and progressive CLL groups, and in mutated or unmutated IGVH CLL groups; data are reported as median and interquartile range (box); whiskers range from min to max. Mann-Whitney test was used to compare groups; **denotes a *p* value ≤ 0.01

In conclusion, differently from previous studies, we calculated VAFs on PBMCs, demonstrating that this is reliable to track CLL evolution. In fact, an increase of a single VAF over time indicates expansion of the clone carrying that NV, regardless of variation in cancer cell fraction. Overall, our study points to a higher genetic dynamics in P-CLLs and suggests that monitoring VAFs of a specific gene panel in PBMCs from sequential samples of a CLL patient may predict disease progression.

## Supplementary information

**Additional file 1: Table S1** Molecular and clinical features of all patients included in the study.

**Additional file 2.** Supplemental Data (Additional files 6 and 7).

**Additional file 3: Table S2** HaloPlex SureDesign Report.

**Additional file 4: Table S3** Genetic variants represented in Fig. 2.

**Additional file 5: Table S4.** Characteristics of patients having few (dNV≤1) versus many (dNV>1) nucleotide variants.

**Additional file 6: Table S6.** SNPs and mutations described by dbSNP and COSMIC databases, respectively, and localized in the regions analyzed by Haloplex SureDesign.

**Additional file 7: Table S7.** SNPs and mutations detected by Haloplex SureDesign Panel.

## Data Availability

DNA CNVs and mutational data are freely available to ArrayExpress database (accession number E-MTAB-8020) and European Nucleotide Archive database (accession number ERP115524). All the other raw data are freely available at the code-hosting platform GitHub (https://github.com/VeroneseVisoneLabs/Genetic-dynamics-in-untreated-CLL-patients-with-either-stable-or-progressive-disease-a-longitudinal).

## Abbreviations

CLL: Chronic lymphocytic leukemia
CN: Copy number
CNV: Copy number variation
dNV: Dynamic nucleotide variant
FTP: First time point
LogR: Log ratio
LTP: Last time point
NV: Nucleotide variant
PBMC: Peripheral blood mononuclear cell
P-CLL: Progressive chronic lymphocytic leukemia
PS: Paired segment
S-CLL: Stable chronic lymphocytic leukemia
VAF: Variant allele frequency

## References

[1] Sutton LA, Rosenquist R (2015). Deciphering the molecular landscape in chronic lymphocytic leukemia: time frame of disease evolution. Haematologica..

[2] Hernandez-Sanchez M, Kotaskova J, Rodriguez AE, Radova L, Tamborero D, Abaigar M (2019). CLL cells cumulate genetic aberrations prior to the first therapy even in outwardly inactive disease phase. Leukemia..

[3] Leeksma AC, Taylor J, Wu B, Gardner JR, He J, Nahas M (2019). Clonal diversity predicts adverse outcome in chronic lymphocytic leukemia. Leukemia..

[4] Rose-Zerilli MJ, Gibson J, Wang J, Tapper W, Davis Z, Parker H (2016). Longitudinal copy number, whole exome and targeted deep sequencing of 'good risk' IGHV-mutated CLL patients with progressive disease. Leukemia..

[5] Hallek M, Cheson BD, Catovsky D, Caligaris-Cappio F, Dighiero G, Dohner H (2008). Guidelines for the diagnosis and treatment of chronic lymphocytic leukemia: a report from the International Workshop on Chronic Lymphocytic Leukemia updating the National Cancer Institute-Working Group 1996 guidelines. Blood..

[6] Mayrhofer M, Viklund B, Isaksson A (2016). Rawcopy: improved copy number analysis with Affymetrix arrays. Sci Rep.

[7] Dohner H, Stilgenbauer S, Benner A, Leupolt E, Krober A, Bullinger L (2000). Genomic aberrations and survival in chronic lymphocytic leukemia. N Engl J Med.

