# Supplementary material for: Genetic dynamics in untreated CLL patients with either stable or progressive disease: a longitudinal study
Source: J Hematol Oncol. 2019 Nov 19;12:114. doi: 10.1186/s13045-019-0802-x (PMC6862808; doi:10.1186/s13045-019-0802-x)
Supplement: Supplementary file 1 — Additional file 1: Table S1 Molecular and clinical features of all patients included in the study. [file 13045_2019_802_MOESM1_ESM.pdf]

**Supplementary table 1. Molecular and clinical features of all patients included in the study.** FTP, first time point; LTP, last time point; WBC, white blood cell; NA, no data available; MBL, Monoclonal B-cells lymphocytosis. IGVH homology mutated = IGVH homology<98%; IGVH homology unmutated = IGVH homology>98%. Zap-70 positive: Zap-70>20%; Zap-70 negative: Zap-70<20%

**Stable CLL**

| ID | Date of Diagnosis | FTP / LTP | Date of First treatment | Date of last follow-up | Time frame (FTP-Date of diagnosis) | Time frame (LTP-FTP) | IGHV homology | Zap-70 | WBC (x10^9/liter) | ALC (% from WBC) | CD19+/CD5+ (% from PBMC) totale | Binet/Rai Stage | Therapy need | Reference | CNV (qPCR) | Mutational analysis | CNV (SNP genotyping) | Affy ID |
|---|---|---|---|---|---|---|---|---|---|---|---|---|---|---|---|---|---|---|
| CLL478 | May-99 | Feb-03 | | Feb-10 | 1375,00 | 1022,00 | mut | neg | 19 | NA | | 0 | No | 1 | yes | yes | yes | A2317-16 |
| | | Nov-05 | | | | | | | 18,7 | 47 | | 0 | | | yes | yes | yes | A2317-15 |
| CLL504 | Dec-83 | Nov-00 | | Jul-05 | 6195,00 | 441,00 | mut | neg | 21 | 82,4 | | NA | No | 1 | yes | yes | yes | A2317-18 |
| | | Jan-02 | | | | | | | 21,4 | 73,6 | | NA | | | yes | yes | yes | A2317-17 |
| CLL514 | Jan-98 | Apr-00 | | Mar-18 | 847,00 | 2931,00 | mut | neg | 11,3 | 74 | | 1 | No | 1 | yes | yes | yes | A2317-20 |
| | | May-08 | | | | | | | 12,7 | 76 | | 2 | | | yes | yes | yes | A231719 |
| CLL534 | Jun-00 | Oct-00 | | Nov-17 | 144,00 | 2432,00 | mut | pos | 10,2 | 41 | | 0 | No | 1 | yes | yes | yes | A2317-22 |
| | | Jun-07 | | | | | | | 14 | 53 | | 1 | | | yes | yes | yes | A2317-21 |
| CLL538 | Jul-99 | Jun-02 | Jul-12 | Feb-18 | 1076,00 | 980,00 | unmut | pos | 17,3 | 63 | | 1 | Yes | 1 | yes | yes | yes | A2317-24 |
| | | Feb-05 | | | | | | | 9,9 | 65 | | 2 | | | yes | yes | yes | A2317-23 |
| LLC003 | Dec-05 | May-11 | | Jul-18 | 1988,00 | | mut | neg | 49,2 | 87,3 | 72,3 | BII | No | 2 | yes | yes | yes | A2317-01 |
| | | May-13 | | | | 741,00 | | | 88 | NA | 94,1 | BII | | | no | yes | yes | A2317-02 |
| | | Oct-15 | | | | 1622,00 | | | 52 | 93,4 | 80,5 | BII | | | yes | no | no | |
| LLC005 | Nov-03 | May-11 | | May-18 | 2755,00 | 660,00 | mut | neg | 50 | 86 | 83,1 | BII | No | 2 | yes | yes | yes | A2317-03 |
| | | Mar-13 | | | | | | | 56 | 91,9 | 68,1 | BII | | | yes | yes | yes | A2317-04 |
| LLC008 | Aug-08 | May-11 | | Jun-17 | 1027,00 | 420,00 | unmut | neg | 15,7 | 65 | 50,9 | A0 | No | 2 | yes | yes | yes | A2317-05 |
| | | Jul-12 | | | | | | | 16 | 72 | 60 | A0 | | | yes | yes | yes | A2317-06 |
| LLC012A | Oct-03 | Feb-12 | | Dec-17 | 3065,00 | 1393,00 | mut | neg | 15 | 55 | 27,6 | A0 | No | 2 | yes | no | no | |
| | | Dec-15 | | | | | | | 9 | 44 | 14,1 | A0 | | | yes | no | no | |
| LLC014 | Sep-09 | Jul-11 | Sep-16 | Aug-18 | 679,00 | | mut | neg | 54 | 82,6 | NA | AII | Yes | 2 | no | yes | no | |
| | | Mar-12 | | | | 259,00 | | | 73 | 77,7 | NA | AII | | | yes | yes | no | |
| | | Oct-14 | | | | 931,00 | | | 70 | 84 | 83,9 | AII | | | yes | no | no | |
| LLC020 | Mar-07 | Oct-11 | | Apr-18 | 1685,00 | 826,00 | unmut | neg | 27 | 94 | 59,5 | A0 | No | 2 | yes | no | no | |
| | | Jan-14 | | | | | | | 28 | 84,4 | 61,1 | AI | | | yes | no | no | |
| LLC021 | May-08 | Oct-11 | | Feb-18 | 1258,00 | 1561,00 | mut | neg | 15 | 60 | 40,7 | A0 | No | 2 | yes | no | yes | A3288-01 |
| | | Jan-16 | | * | | | | | 29 | 65,5 | 53,9 | A0 | | | yes | no | yes | A3288-02 |
| LLC031 | Sep-09 | Nov-11 | | | 819,00 | 1267,00 | mut | neg | 15 | 71 | 63,3 | A0 | No | 2 | yes | no | no | |
| | | May-15 | | | | | | | 9 | 79,7 | 64,3 | A0 | | | yes | no | no | |
| LLC035 | Oct-00 | Dec-11 | | Sep-18 | 4084,00 | 532,00 | mut | neg | 34,3 | 84 | 84,8 | BII | No | 2 | no | yes | yes | A2317-27 |
| | | May-13 | | | | | | | 23 | 86 | 78,8 | BII | | | no | yes | yes | A2317-28 |
| LLC039 | Sep-05 | Jan-12 | | May-18 | 2322,00 | 1519,00 | mut | neg | 68 | 89,7 | 95,5 | BII | No | 2 | yes | no | no | |
| | | Mar-16 | | | | | | | 107 | 90 | 89,5 | BII | | | yes | no | no | |
| LLC052 | Jan-10 | Mar-12 | | Oct-17 | 795,00 | 378,00 | mut | neg | 29 | 88 | 66,2 | AII | No | 2 | no | yes | no | |
| | | Mar-13 | | | | | | | 33 | 90 | 87,7 | BII | | | no | yes | no | |
| LLC056 | Aug-05 | Mar-12 | | Nov-17 | 2416,00 | 1372,00 | mut | neg | 29 | 75,2 | 48,9 | BII | No | 2 | yes | no | no | |
| | | Dec-15 | | | | | | | 33 | 75 | 30,3 | BII | | | yes | no | no | |
| LLC066 | Nov-11 | Sep-12 | | Jun-17 | 329,00 | 1176,00 | mut | neg | 12.8 | 62 | 31,8 | A0 | No | 2 | yes | no | yes | A3288-07 |
| | | Dec-15 | | | | | | | 15.7 | 76,4 | 65,6 | AI | | | yes | no | yes | A3288-08 |
| LLC086 | Mar-10 | May-13 | | Apr-18 | 1184,00 | 966,00 | unmut | neg | 10 | 70,1 | 37,2 | A0 | No | 2 | yes | no | no | |
| | | Jan-16 | | | | | | | 12 | 74,5 | 48,8 | A0 | | | yes | no | no | |
| | | | | median | 1258,00 | 980,00 | | | | | | | | | | | | |

**Progressive CLL**

| ID | Date of Diagnosis | FTP / LTP | Date of First treatment | Date of last follow-up | Time frame (FTP-Date of diagnosis) | Time frame (LTP-FTP) | IGHV homology | Zap-70 | WBC (x10^9/liter) | ALC (% from WBC) | CD19+/CD5+ (% from PBMC) | Binet/Rai Stage | Therapy need | Reference | CNV (qPCR) | Mutational analysis | CNV (SNP genotyping) | Affy ID |
|---|---|---|---|---|---|---|---|---|---|---|---|---|---|---|---|---|---|---|
| CLL200 | Dec-97 | Feb-01 | Jul-13 | | 1158,00 | 754,00 | mut | neg | 43,3 | 93 | | 1 | yes | 1 | yes | yes | yes | A2317-07 |
| | | Feb-03 | | | | | | | 63,2 | 88 | | 2 | | | yes | yes | yes | A2317-08 |
| CLL206 | Feb-94 | Jul-00 | | Apr-13 | 2368,00 | 2660,00 | mut | neg | 94,5 | 85 | | 1 | No | 1 | yes | yes | no | |
| | | Nov-07 | | | | | | | 170,5 | 91 | | 2 | | | yes | yes | no | |
| CLL211 | Jul-99 | Jan-00 | Oct-11 | | 203,00 | 1230,00 | mut | neg | 28,8 | 86 | | 0 | yes | 1 | yes | yes | yes | A2317-09 |
| | | Jun-03 | | | | | | | 126 | 93 | | 1 | | | yes | yes | yes | A2317-10 |
| CLL221 | Dec-00 | Jan-01 | Apr-06 | Apr-13 | 8,00 | 1801,00 | unmut | pos | 30,8 | 65 | | 1 | Yes | 1 | yes | yes | yes | A2317-11 |
| | | Dec-05 | | | | | | | 439 | 89 | | 2 | | | yes | yes | yes | A2317-12 |
| CLL227 | Dec-03 | Apr-05 | Jan-07 | Dec-08 | 508,00 | 605,00 | unmut | pos | 21,5 | 82 | | NA | Yes | 1** | yes | yes | no | |
| | | Dec-06 | | | | | | | 152 | 92 | | NA | | | yes | yes | no | |
| CLL233 | Oct-03 | Oct-04 | Feb-07 | May-18 | 391,00 | 805,00 | unmut | pos | 35,1 | 71 | | NA | Yes | 1*** | yes | yes | no | |
| | | Jan-07 | | | | | | | 122,5 | 91 | | NA | | | yes | yes | no | |
| CLL237 | Feb-04 | Nov-05 | Nov-15 | Oct-17 | 639,00 | 426,00 | unmut | pos | 54,9 | 86 | | 2 | Yes | 1 | yes | yes | yes | RV-237 |
| | | Jan-07 | | | | | | | 112 | 89 | | 2 | | | yes | yes | yes | RV-241 A |
| CLL242 | Jul-01 | Mar-06 | Nov-08 | Dec-08 | 1709,00 | 393,00 | mut | neg | 135 | 94 | | 2 | Yes | 1 | yes | yes | no | |
| | | Apr-07 | | | | | | | 209 | 98 | | 4 | | | yes | yes | no | |
| CLL248 | Jun-01 | Dec-01 | Jun-04 | Aug-05 | 187,00 | 890,00 | unmut | pos | 43,1 | 79 | | NA | Yes | | yes | yes | no | |
| | | May-04 | | | | | | | 338 | 88 | | NA | | | yes | yes | no | |
| CLL251 | Jun-01 | Sep-01 | Oct-06 | | 103,00 | 1455,00 | unmut | neg | 25,3 | 83 | | NA | Yes | 1 | yes | yes | yes | RV-251 |
| | | Sep-05 | | | | | | | 36,7 | 72 | | NA | | | yes | yes | yes | RV-254 |
| CLL255 | Jan-01 | Aug-01 | Feb-04 | | 224,00 | 890,00 | mut | ND | 14,8 | 56 | | NA | Yes | 1 | no | no | yes | RV-255 A |
| | | Feb-04 | | | | | | ND | 127,8 | 78 | | NA | | | no | no | yes | RV-258 |
| CLL259 | Jun-00 | Sep-02 | Apr-05 | | 833,00 | 936,00 | mut | neg | 87,5 | 88 | | NA | Yes | 1 | no | yes | yes | RV-259 |
| | | Apr-05 | | | | | | | 306 | 99 | | NA | | | no | yes | yes | RV-414 A |
| CLL262a | Jan-97 | Jun-03 | Apr-07 | Aug-10 | 2357,00 | 1248,00 | mut | neg | 14 | 68 | | 0 | Yes | 1 | yes | no | no | |
| | | Nov-06 | | | | | | | 207.2 | 88 | | 1 | | | yes | no | no | |
| CLL265 | Oct-01 | May-03 | Jul-07 | Jun-08 | 606,00 | 930,00 | unmut | pos | 147,5 | 94 | | 0 | Yes | 1 | yes | no | yes | RV-265 |
| | | Dec-05 | | | | | | | 201 | 81 | | 2 | | | yes | no | yes | RV-268 |
| CLL400t | Oct-99 | Feb-01 | | Feb-05 | 515,00 | 1455,00 | ND | ND | 55 | 66 | | NA | No | | no | yes | no | |
| | | Feb-05 | | | | | ND | ND | 165,4 | 93 | | NA | | | no | yes | no | |
| CLL408 | Jan-99 | Oct-02 | | Feb-14 | 1390,00 | 1245,00 | mut | neg | 48,7 | 91 | | 0 | No | 1 | yes | yes | no | |
| | | Mar-06 | | | | | | | 167.4 | 92 | | 2 | | | yes | yes | no | |
| CLL416 | Feb-92 | Jan-00 | May-04 | Aug-09 | 2891,00 | 1456,00 | mut | neg | 65,2 | 85 | | NA | Yes | 1 | yes | yes | yes | RV-416 |
| | | Jan-04 | | | | | | | 210 | 93 | | 2 | | | yes | yes | yes | RV-417 |
| CLL422 | Mar-03 | Feb-04 | | Oct-09 | 360,00 | 973,00 | mut | neg | 53,6 | 90 | | NA | No | 1 | no | no | yes | RV-422 |
| | | Oct-06 | | | | | | | 157,6 | 90 | | NA | | | no | no | yes | RV-423 |
| CLL523 | Jan-98 | Sep-99 | Aug-04 | Jan-05 | 616,00 | 1419,00 | unmut | pos | 16 | 74 | | 3 | Yes | 1 | no | yes | no | |
| | | Jul-03 | | | | | | | 6,4 | 63 | | 4 | | | no | yes | no | |
| LLC004 | Jul-08 | May-11 | Apr-14 | May-16 | 1043,00 | 1043,00 | unmut | pos | 41,3 | 80 | NA | BII | No | 2 | yes | no | no | |
| | | Mar-14 | | | | | | | 48 | 87,5 | 83,5 | BII | | | yes | no | no | |
| LLC034 | Sep-10 | Dec-11 | Apr-15 | Sep-18 | 456,00 | | mut | neg | 24 | 55,2 | 20,6 | A0 | yes | 2 | no | yes | yes | A2317-25 |
| | | Oct-12 | | | | | | | 24,2 | 81,1 | 68,8 | BII | | | yes | no | no | |
| | | May-13 | | | | 545,00 | | | 54 | 81 | 88,3 | BII | | | no | yes | yes | A2317-26 |
| | | Apr-14 | | | | 525,00 | | | 60,6 | 92,4 | 80,4 | BII | | | yes | no | no | |
| LLC045 | May-05 | Jan-12 | Mar-17 | Jun-18 | 2466,00 | 1149,00 | unmut | neg | 51 | 59 | 92,5 | BII | yes | 2 | yes | no | no | |
| | | Mar-15 | | | | | | | 60 | 83 | 89,3 | CIV | | | yes | no | no | |
| LLC047 | Apr-10 | Feb-12 | | Sep-18 | 684,00 | 1435,00 | mut | neg | 15.3 | 55 | 36,1 | A0 | No | 2 | yes | no | yes | A3288-05 |
| | | Jan-16 | | | | | | | 24.5 | 73,5 | 58,9 | BII | | | yes | no | yes | A3288-06 |
| LLC070 | Oct-07 | Oct-12 | Apr-13 | Oct-18 | 1835,00 | 120,00 | unmut | pos | 72,7 | 83 | 87,9 | BII | Yes | 2 | no | no | yes | A3288-09 |
| | | Feb-13 | | | | | | | 134 | 87 | 86,4 | BII | | | no | no | yes | A3288-10 |
| LLC081 | Jun-11 | Jan-13 | Feb-14 | Oct-18 | 608,00 | 379,00 | mut | neg | 72 | 87 | 76,9 | BII | Yes | 2 | no | no | yes | A3288-11 |
| | | Feb-14 | | | | | | | 113,6 | 78 | 93,9 | BII | | | no | no | yes | A3288-12 |
| LLC088 | Jun-12 | Jun-13 | Apr-15 | Oct-18 | 368,00 | 609,00 | mut | ND | 47 | 80 | 19,4 | BII | Yes | 2 | yes | no | no | |
| | | Feb-15 | | | | | | ND | 88 | 87,9 | 46,2 | BII | | | yes | no | no | |
| LLC098 | Oct-13 | Feb-14 | Jun-14 | Sep-18 | 147,00 | 112,00 | unmut | neg | 38,66 | 77,6 | 84,8 | BII | Yes | 2 | no | no | yes | A3288-13 |
| | | Jun-14 | | **** | | | | | 85,99 | 75 | 92,9 | BII | | | no | no | yes | A3288-14 |
| LLC104 | Nov-11 | Jun-14 | | Sep-18 | 947,00 | 627,00 | mut | neg | 25,5 | 77,2 | 70,3 | AI | No | 2 | yes | no | no | |
| | | Feb-16 | | | | | | | 50,3 | 63,6 | 87,8 | BII | | | yes | no | no | |
| LLC110 | Jun-12 | Dec-14 | | Oct-18 | 928,00 | 441,00 | mut | neg | 38 | 65,8 | 85,7 | A0 | No | 2 | yes | no | no | |
| | | Mar-16 | | | | | | | 61 | 85,9 | 90 | BII | | | yes | no | no | |
| LLC76-9 | | Jan-14 | | | | | | | | | 84,2 | NA | | | yes | no | no | |
| | | Jun-14 | | | | | | | | | 88 | NA | | | yes | no | no | |
| | | | | median | 616,00 | 910,00 | | | | | | | | | | | | |

| | |
|---|---|
| 1 | Patient included in the study Visone et al. (2011) |
| 2 | Patient recruited at Policlinico Gemelli, Rome, Italy |
| * | LLC021 died in march 2018 (not CLL-related) |
| ** | CLL227 is the previous time point of 228 in Visone et al.(2011) |
| *** | CLL233 is the previous time point of 234 in Visone et al. (2011) |
| **** | LLC098 died in september 2018 (CLL-related) |
