# Supplementary material for: Genetic dynamics in untreated CLL patients with either stable or progressive disease: a longitudinal study
Source: J Hematol Oncol. 2019 Nov 19;12:114. doi: 10.1186/s13045-019-0802-x (PMC6862808; doi:10.1186/s13045-019-0802-x)
Supplement: Supplementary file 2 — Additional file 2. Supplemental Data (Additional files 6 and 7). [file 13045_2019_802_MOESM2_ESM.docx]

**Supplemental Data**

# Genetic dynamics in untreated CLL patients with either stable or progressive disease: a longitudinal study

Alice Ramassone^1,2*^, Andrea D’Argenio^1*^, Angelo Veronese^1,2*^, Alessio Basti^3,4^, Shimaa H.A. Soliman^1,2^, Stefano Volinia^5^, Cristian Bassi^5^, Sara Pagotto^1,8^, Manuela Ferracin^6^, Laura Lupini^5^, Elena Saccenti^5^, Veronica Balatti^7^, Felice Pepe^1,8^, Laura Z. Rassenti^9,10,11^, Idanna Innocenti^12^, Francesco Autore^12^, Laura Marzetti^3,4^, Renato Mariani-Costantini^1,8^, Thomas J. Kipps^9,10,11^, Massimo Negrini^5^, Luca Laurenti^12^ and Rosa Visone^1, 8§^

^1^ Unit of General Pathology, Center for Advanced Studies and Technology (CAST), University G. d’Annunzio Chieti-Pescara, Chieti, Italy

^2^ Department of Department of Medicine and Aging Science, University G. d’Annunzio Chieti-Pescara, Chieti, Italy

^3^ Department of Neuroscience, Imaging and Clinical Sciences, University G. d’Annunzio Chieti-Pescara, Chieti, Italy;

^4^ Institute for Advanced Biomedical Technologies (ITAB), University G. d’Annunzio Chieti-Pescara, Chieti, Italy;

^5^ Department of Morphology, Surgery and Experimental Medicine, University of Ferrara, Ferrara, Italy

^6^ Department of Experimental, Diagnostic and Specialty Medicine, University of Bologna, Bologna, Italy

^7^ Department of Cancer Biology and Genetics and Comprehensive Cancer Center at the Wexner Medical Center, The Ohio State University, Columbus, OH, USA;

^8^ Department of Medical, Oral and Biotechnological Sciences, University G. d’Annunzio Chieti-Pescara, Chieti, Italy

^9^ Department of Medicine, Moores Cancer Center, University of California at San Diego, La Jolla, CA, USA

^10^ Chronic Lymphocytic Leukemia Research Consortium, San Diego, CA, USA

^11^ The CLL Research Consortium (National Institutes of Health PO1-CA81534)

^12^ Fondazione Policlinico Universitario A Gemelli IRCCS, Rome, Italy

* These authors contributed equally to this work.

^§^ Corresponding author:

Rosa Visone, PhD

Department of Medical, Oral and Biotechnological Sciences

University G. d'Annunzio

Via dei Vestini 31, 66100 Chieti, Italy

Tel: +39-0871-541498

Email: [r.visone@unich.it](mailto:r.visone@unich.it)

**Methods**

**Patients and samples**

Samples were provided by Policlinico Agostino Gemelli (Rome, Italy) and biobanks of the CLL Research Consortium (San Diego, USA) upon written informed consent in accordance with the Declaration of Helsinki. The institutional review board of the University of California, San Diego (171884CX) and of the Fondazione Policlinico Agostino Gemelli (P/948/CE/2011) approved the research protocol. Samples and clinical data were collected from 49 patients at two time points (first time point, FTP; last time point, LTP) between 2011 and 2016 (at Policlinico Agostino Gemelli) and between 2000 and 2009 (at the CLL Consortium). All patients were untreated at samplings (**SuppTable01**). At each time point the diagnosis of stable or progressive CLL was established by the clinician according to the criteria defined during the International Workshop on Chronic Lymphocytic Leukemia (1). At FTP, all patients had stable disease (S-CLL); at LTP, 30/49 had developed progressive disease (P-CLL). The median time of sampling was 980 days for S-CLLs and 910 days for P-CLLs. Samples were chosen based on similar median frame times between the progressive and stable cohorts and on material availability.

Genomic DNA was extracted from PBMCs using either PureLink Genomic DNA Mini Kits (Thermo Fisher Scientific), for genotyping and NGS, or lysis in 0.6% SDS, 10mM EDTA, 10mM Tris-HCl, 200 mg/ml proteinase K, followed by phenol/chloroform/isoamyl extraction for qPCR. DNA samples quality was checked by gel electrophoresis and quantity by spectrophotometer analysis (Nanodrop, Thermo Fisher Scientific); samples with poor quality/quantity were excluded from the analyses.

**Genome-wide CNV analysis**

DNAs from 11 patients with stable CLL and from 15 patients with progressive CLL were genotyped using Genome-Wide Human SNP Arrays 6.0 (Affymetrix) at two time points: first time point (FTP) and last time point (LTP). Copy number variations (CNVs) were identified using the Rawcopy package (2), which allows to measure DNA abundance by performing a segmentation of the genome in regions with different log ratios (LogR). Data from sex chromosomes were excluded from analysis. SNP Array data were deposited on ArrayExpress database (accession number E-MTAB-8020).

*Segmentation of the genome*

For single-subject longitudinal analysis, we introduced a novel segmentation strategy, designated Paired Segmentation. To obtain segments with the same breakpoints in FTP and LTP, the new Paired Segments (PSs) set of breakpoints was defined as the set containing all the breakpoints in the FTP and LTP segmentations, with no repetitions (**Fig. S1**). Specifically, by considering $\left\{ b_{{FTP}_{i}} \right\}_{i=1,\ldots,N}$as all the FTP breakpoints and $\left\{ b_{{LTP}_{i}} \right\}_{i=1,\ldots,M}$as all the LTP breakpoints, the PS breakpoints were defined as $\left\{ b_{\sigma_{i}} \right\}_{i=1,\ldots,K}$ where $K$ is the largest number of different breakpoints obtained from the FTP and LTP sets, $b_{\sigma_{i}}\leq b_{\sigma_{i+1}}$ and $\forall i=1,\ldots,K$∃$j$ such that $b_{\sigma_{i}}=b_{{FTP}_{j}}˅$ $b_{{LTP}_{j}}.$ A Global Segmentation approach was defined to perform an analysis among patients. The approach was the same used to obtain an individual PS. Indeed, the breakpoints of the Global Segments (GSs) were defined as all the different breakpoints of the individual PS for the 27 patients (**Fig.S1**). Code is available upon request.

*Definition of aberrant loci*

As previously described by Van Loo et al., in a diploid sample the log ratio (LogR) value for a genomic locus can be written as $LogR:=\gamma{{log}_{2}(\alpha}/{\beta)}$, where $\alpha$ denotes copy number of the locus, $\beta$ ploidy and $\gamma$ is an array-specific parameter (3). In our experiments $\gamma$was equal to $0.547$. Furthermore, we assumed $\beta=2$, since CLL samples have a ploidy approximately equal to 2 (4).

In patient-derived samples composed of $f$ cancer cells and $\left( 1-f \right)$ normal (diploid) cells, the copy number $\alpha$ is the result of the copy number in both cancer and normal cells. Specifically, $\alpha=2\left( 1-f \right)+kf$, where 2 is the copy number of the normal cells (3, 5), and *k* is the average copy number of the cancer cells.

We defined the aberrant loci with two different approaches: a) using a threshold on LogR; b) using a threshold on *k*.

1. *Threshold on LogR*

If the percentage of cancer cells was unknown, $LogR={\gamma log}_{2}\left( \frac{2\left( 1-f \right)+kf}{2} \right)$, where *k* was fixed equal to 3 or 1 to denote DNA amplification or deletion, and *f* varied from 1 to 100%. We introduced two sets of thresholds on the LogR as a function of *f*, one set for DNA amplification (LogR_A_) and one set for DNA deletion (LogR_D_):

$${{LogR}_{A}=\left\{ {\gamma log}_{2}\left( \frac{2\left( 1-f \right)+3f}{2} \right) \right\}}_{f=0.01,\ldots,1}$$

$${{LogR}_{D}=\left\{ {\gamma log}_{2}\left( \frac{2\left( 1-f \right)+f}{2} \right) \right\}}_{f=0.01,\ldots,1}$$

Based on LogR value in the FTP and LTP, each paired segment showing an aberrant copy number in at least one time point (FTP or LTP), was sorted into one of the following classes (**Fig.S2**):

- **No change of** **aberrations**: both in FTP and in LTP $LogR\leq{LogR}_{D} or LogR\geq{LogR}_{A}$.
- **Acquisition of** **aberrations**: in FTP ${LogR}_{D}<LogR<{LogR}_{A}$, while in LTP $LogR\leq{LogR}_{D} or LogR\geq{LogR}_{A}$.
- **Loss of** **aberrations**: in FTP $LogR\leq{LogR}_{D} or LogR\geq{LogR}_{A}$, while in LTP ${LogR}_{D}<LogR<{LogR}_{A}$.

1. *Threshold on k*

If the percentage *f* of cancer cells was known, $k=\frac{2}{f}(2^{{logR}/\gamma}+f-1)$. We defined a locus as aberrant if $k_{A}\geq2.5$ (DNA amplification), or if $k_{D}\leq1.5$ (DNA deletion). Based on *k* value in the FTP and LTP, each paired segment showing an aberrant copy number in at least one time point (FTP or LTP), was sorted into one of the following classes (**Fig.S7**):

- **No change of** **aberrations**: both in FTP and in LTP $k\leq k_{D} or k\geq k_{A}$.
- **Acquisition of** **aberrations**: in FTP $k_{D}<k<k_{A}$, while in LTP $k\leq k_{D} or k\geq k_{A}$ .
- **Loss of** **aberrations**: in FTP $k\leq k_{D} or k\geq k_{A}$, while in LTP $k_{D}<k<k_{A}$.

*Definition of the segment’s slope*

The slope was assessed on the LogR of the paired segments and defined as the difference between the LogR in the LTP and FTP, $\Delta{LogR}_{LTP,FTP}$, divided by the number of days between the two acquisitions, ${\Delta t}_{LTP,FTP}$:

$$slope=\frac{\Delta{LogR}_{LTP,FTP}}{{\Delta t}_{LTP,FTP}}$$

**CNV analysis by qPCR**

DNA from a total of 18 and 22 patients with either stable or progressive disease were analysed by qPCR on a 7900HT Real-Time PCR system using the Universal ProbeLibrary System Technology (Roche). Briefly, 10 ng of genomic DNA was amplified with the primers detailed in **SuppTable05**, a fluorescent probe and an internal reference control (TaqMan RNase P assay, Applied Biosystem). Each sample was analysed in triplicate for the gene and the internal normalizer RNaseP to obtain 3 values of ΔCt ($2^{-(gene FAM dye Ct-RNaseP VIC dye Ct)}$). The average ΔCt for each triplicate was calculated and then normalized to a calibrator (two DNA samples from PBMCs of healthy blood donors) to determine ΔΔCt.

**Mutational analysis of CLL-related genes**

Specific regions of 27 genes mutated in CLL were sequenced by the Ion PGM System (Thermo Fisher) in 11 and 17 CLLs with either stable or progressive disease. The sequenced genes included *BCOR*, *EGR2*, *HIST1H1E*, *ITPKB*, *KRAS*, *MED12*, *NRAS*, *RIPK1*, *SAMHD1, ATM*, *BIRC3*, *BRAF*, *CHD2*, *DDX3X*, *DDX3Y, FBXW7*, *KIT*, *KLHL6*, *MAPK1*, *MYD88*, *NOTCH1*, *PIK3CA, POT1*, *SF3B1*, *TP53*, *XPO1* and *ZMYM3*, which were previously identified as mutated in CLL studies (**SuppTable02**) (4, 6-11). Mutational analysis was performed by next generation sequencing. Briefly, HaloPlex Target Enrichment kits (Agilent Technologies, Santa Clara, CA, USA) were used to produce libraries of the targeted genomic DNA regions starting from genomic DNA of PBMCs. Diluted libraries were linked to Ion Sphere Particles, clonally amplified and enriched by emulsion PCR on an Ion OneTouch System (Life Technologies). Enriched template-positive Ion Sphere Particles were loaded onto Ion chips and sequenced using the Ion PGM System. Sequencing data were aligned to the human reference genome (GRCh37). Sequence variants were identified using Torrent Suite 3.4 and Variant Caller plugin 3.4.4, as previously described (12). To avoid false positives, variants with coverage <100 or single nucleotide variants found within ≥4 repeats of the same nucleotide were excluded; variants with frequency <5% were also excluded. DbSNP and COSMIC databases were inspected to identify all the SNPs and mutations interrogated (**SuppTable06**) registering 1021 NVs (**SuppTable07**) with an average of 18.23 NVs per sample. Some of the identify mutations were also validated by Sanger sequencing (**Fig.S8**). Variants that changed more than the 20% between the two clinical time points were considered for further analysis. Sequencing data were deposited on European Nucleotide Archive database (accession number ERP115524).

**Sanger sequencing**

The DNA regions of interest were amplified with primers detailed in **SuppTable05** and sequenced using the BigDye Terminator v3.1 Cycle Sequencing Kit (Life Technologies, Waltham, MA, USA) according to the manufacturer’s specification, with an ABI3130xl Genetic Analyzer (Life Technologies).

**Statistical analysis**

Statistical analyses were performed by using GraphPad Prism 6.01 (GraphPad Software), or MATLAB R2014a (Mathworks). Normality of distributions was assayed by D’Agostino & Pearson or Kolmogorov-Smirnov normality tests. Based on the normality test, Mann-Whitney U-test or unpaired t-test and Wilcoxon matched-pairs test or paired t-test was used as indicated in figure legends. For multiple comparison, Bonferroni correction was used. Fisher’s exact test was used to evaluate the associations between dichotomous categorical variables. Kaplan-Meier method was used to estimate time from first time point, or from diagnosis, to initial treatment or last follow-up and significance was assessed by log-rank test; cut-off value was calculated by the ROC curve. The use of mean± standard deviation or median with range is indicated in figure legends. Statistical tests were two-sided, * denotes a p-value≤0.05, ** denotes a p-value≤0.01 and *** denotes a p-value≤0.001.

**Additional data:**

**Validation of the used method to define the aberrant loci**

To validate the method used to define the aberrant loci, we used a subset of patients, including 6 S-CLLs and 5 P-CLLs, for which the percentage of cancer cell *f* was known. We defined as aberrant the loci showing amplification or deletion of the DNA. Firstly, we used *f* (the percentage of CD19+/CD5+ in PBMC) to infer the copy number *k* of each locus and we fixed two thresholds on *k*, *k_A_,* and *k_D_*, to indicate DNA amplification or deletion, respectively (**Fig.S7**). Secondly, as reported in the main text and in **Fig.1**, we identified the aberrant loci by varying *f*, thus assuming that the exact percentage of cancer cells was unknown. In this case, we used two sets of thresholds on log ratio (LogR) value, which depends on *f* and *k,* one for amplifications (𝐿𝑜𝑔𝑅_𝐴_), and one for deletions (𝐿𝑜𝑔𝑅_𝐷_) (**Fig.S2**). We thus compared the two methods and we obtained comparable results between the same subset of patients both analysing the percentage of aberrant loci (**Fig.S3**) and their slopes (**Fig.S4**).

**Results of global segmentation analysis**

We sought to identify if there are specific genomic aberrations, which could expand faster in P-CLL patients. To this end, we used a Global Segmentation approach to define common segments among all patients (**Fig.S1**). Then, we calculated the slopes of these segments and investigated possible differences between the S-CLL and P-CLL groups. We did not find any significant differences between the two cohorts.

**Chromosomal abnormalities of prognostic value and genomic regions including microRNAs do not distinguish P-CLLs from S-CLLs**

We used qPCR to assess three chromosomal abnormalities of prognostic value (11q deletion, trisomy 12 and 17p deletion) (13) in a total of 18 stable and 22 progressive patients. We confirmed that trisomy 12 was more common among progressive patients (11.8% vs 28.6% in S-CLLs and P-CLLs, respectively); furthermore, using probes either on arm 12p (ASUN) or arm 12q (HAL), we found that in the patient CLL221, the clone having trisomy 12 increased over disease progression. Using probe on 17p arm (TP53) we also identified, in the patient CLL227, the increase of the clone carrying 17p deletion during disease progression (**Fig.S5**). Overall the investigated aberrations did not significantly change over time between the two groups.

Using LogR data to assess CNVs, we also investigated chromosomal regions comprising miRNAs known to be deregulated in CLL (14-16) in 11 S-CLL and 15 P-CLL patients. According to del(13q14) data, the region encompassing the *miR-15a/16-1* cluster resulted deleted both in S-CLLs and in P-CLLs, and differences between FTP and LTP were not significant in either groups (**Fig.S9**).

**
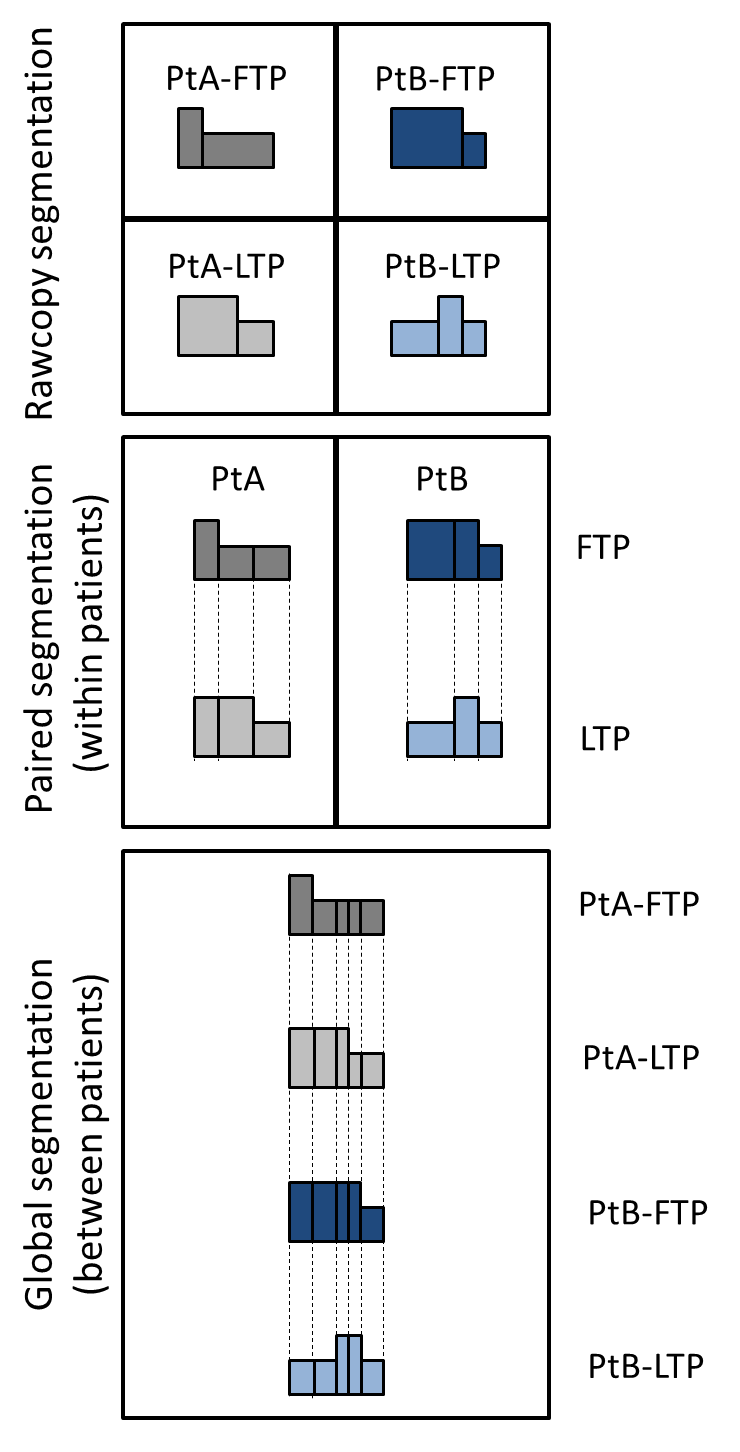
**

**Fig.S1: Graphical representation of the strategy to identify Paired Segments (PSs) and Global Segments (GSs).** Pt: Patient; FTP: First Time Point; LTP: Last Time Point.


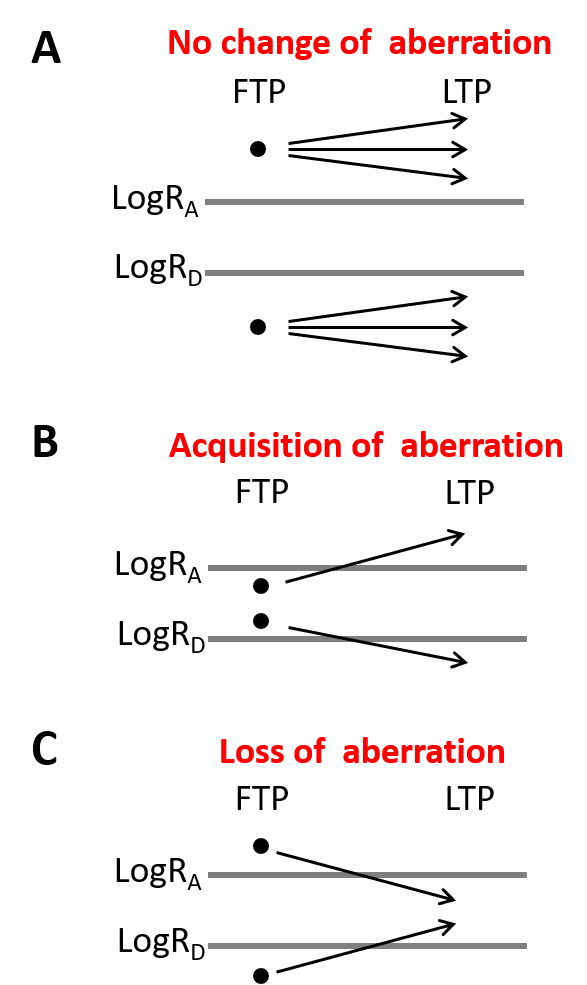


**Fig.S2: Classes assignment of the PS segments, based on their LogR at the FTP and LTP.** A segment whose LogR value exceeds either the LogR_A_ or the LogR_D_ thresholds (for an amplification or deletion, respectively) has been considered as aberrant. A segment, which is aberrant in at least one of the two temporal acquisitions, has been assigned to one of these three classes: A) No change of aberration: the segment is aberrant in both FTP and LTP; B) Acquisition of aberration: the segment is aberrant only in the LTP; C) Loss of aberration: the segment is aberrant only in the LTP. FTP: First Time Point; LTP: Last Time Point.


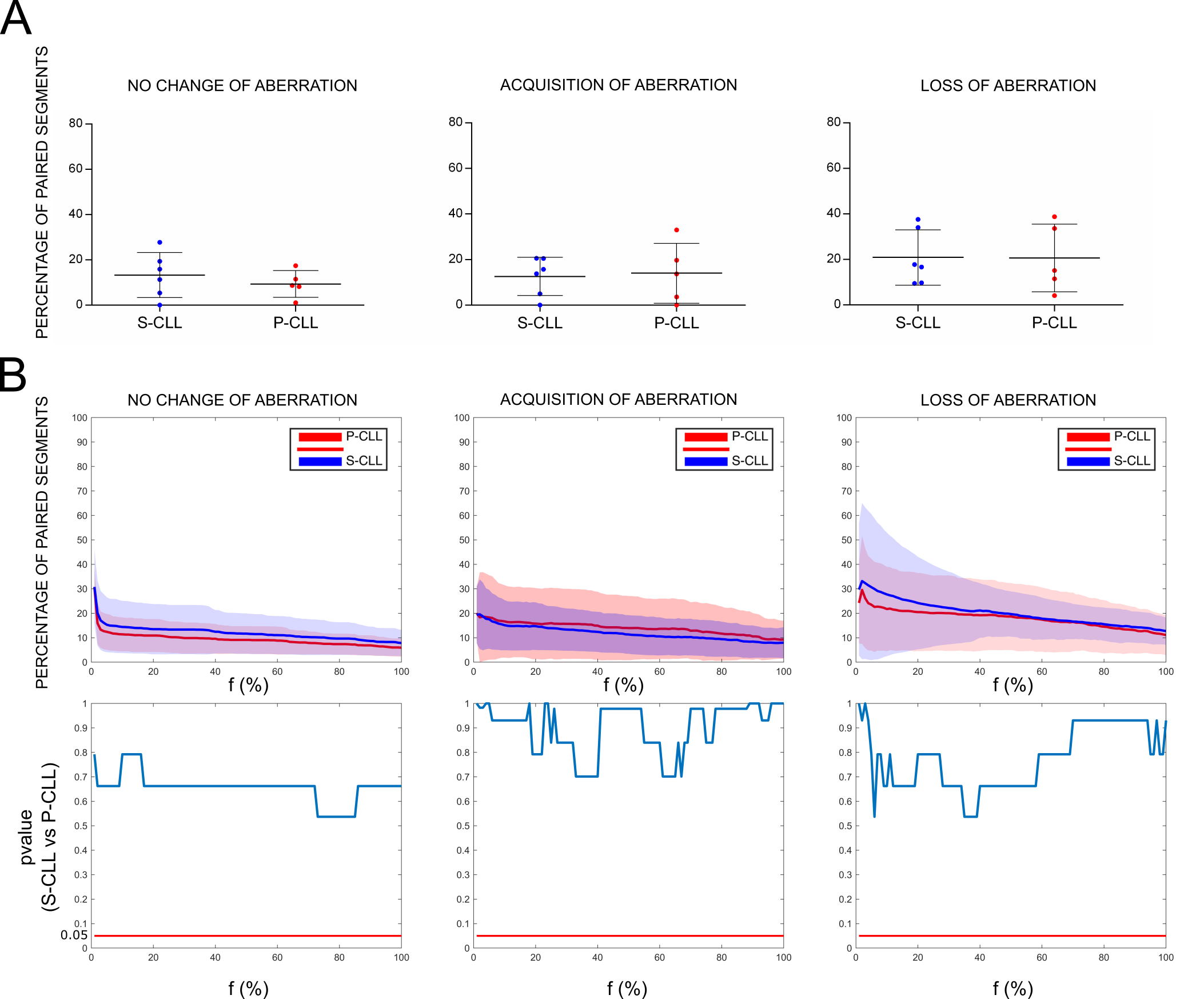


Fig.S3 Validation of longitudinal analysis of the percentage of paired segments (PSs) in samples from 6 stable and 5 progressive patients. A) The percentage of PSs of the three classes (no change, acquisition and loss of aberration) was calculated as a function of *k* (copy number in cancer cells*)*; *k* of each locus was inferred by the percentage of cancer cell (the percentage of CD19+CD5+ cells in PBMC) *f*. Mean ± standard deviation was reported; Mann-Whitney U-test was used to compare S-CLLs and P-CLLs. B) The percentage of PS of the three classes as shown as mean (solid line) and standard deviation (shade) by varying *f*. Red colors indicate the P-CLLs; Blue the S-CLLs.p values’ graphs (lower panel) report the Mann-Whitney U-test for each *f,* significance was defined as P<0.050.


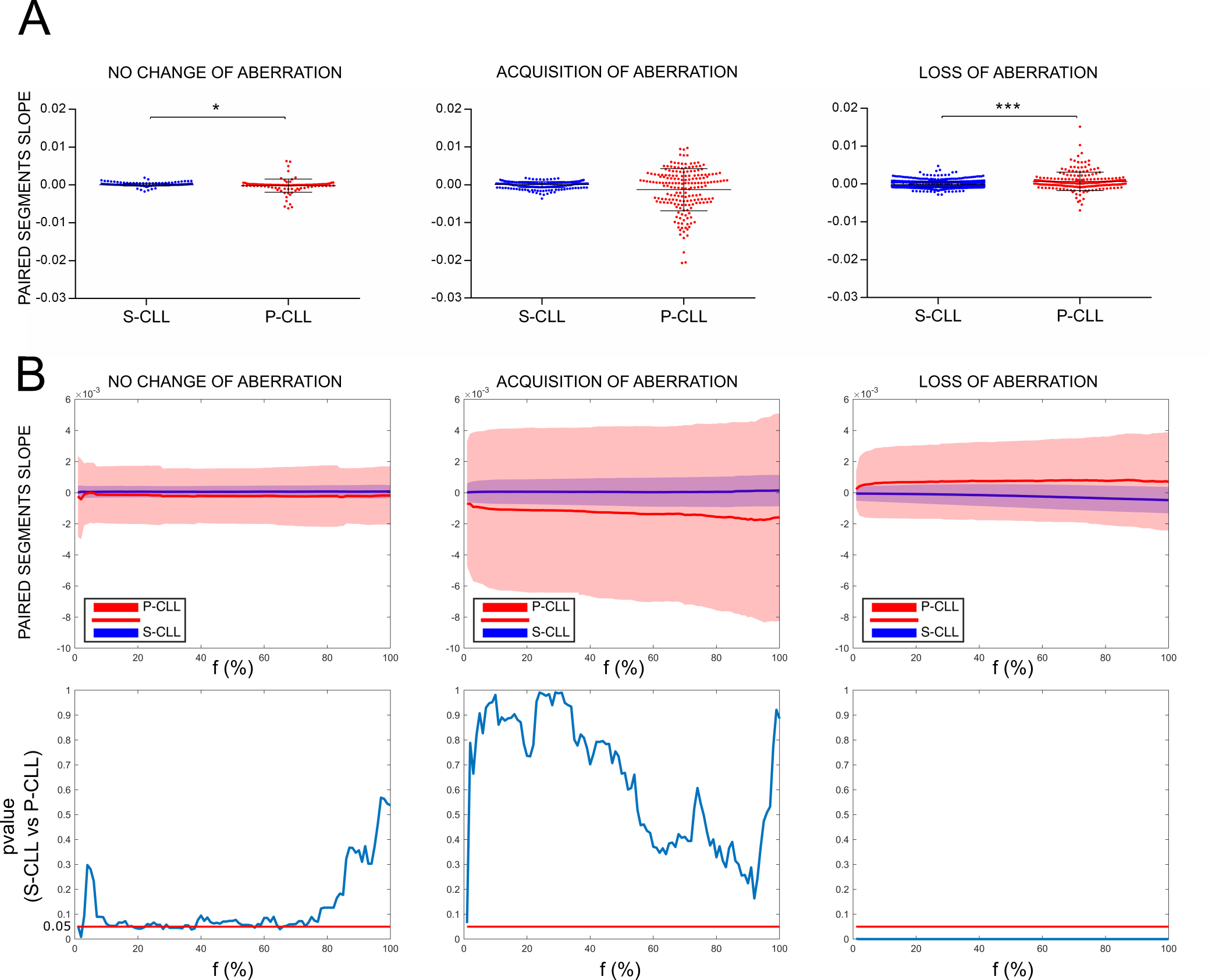


Fig.S4: Validation of longitudinal analysis of the distribution of the slopes of the paired segments (PSs) in samples from 6 stable and 5 progressive patients. A) The slopes $\mathbf{(}{{\boldsymbol{\Delta}\boldsymbol{LogR}}_{\boldsymbol{LTP}\mathbf{,}\boldsymbol{FTP}}}/{{\boldsymbol{\Delta}\boldsymbol{t}}_{\boldsymbol{LTP}\mathbf{,}\boldsymbol{FTP}}\mathbf{)}}$ of the PSs are shown based on no change, acquisition or loss of aberration as a function of *k* (copy number in cancer cells*)*; *k* of each locus was inferred by the percentage *f* of cancer cell (percentage of CD19+CD5+ cells in PBMC). Mean ± standard deviation was reported; Mann-Whitney U-test was used to compare S-CLLs and P-CLLs. B) The slopes of all the paired segments of the three classes are shown as mean (solid line) and standard deviation (shade) by varying *f*. Red colors indicate the P-CLLs; Blue the S-CLLs. p values’ graphs (lower panel) report the mann-whitney U-test for each *f,* significance was defined as P<0.050.





**Fig.S5: Longitudinal analysis of copy number variations for genes belonging to chromosomal regions 11q23, 12p11.23, 12q23.1, 17p13.1.** The allelic quantitation was assessed by qPCR at the 11q deletion (ATM; 11q23), 17p deletion (TP53; 17p13.1) and trisomy 12 (ASUN, 12p11.23, HAL, 12q23.1); data were normalized to the endogenous references RNaseP with the 2^−Δct^ method. The average ΔCt for each triplicate was then normalized to a calibrator (two DNA samples from PBMCs of healthy blood donors) to determine ΔΔCt. A relative allelic quantitation lower than 0.75 denotes DNA deletion, while a relative allelic quantitation higher than 1.25 denotes DNA amplification. Each floating bar represents median with min to max of values obtained from technical triplicate. Two-sample t-test was uses to compare the ΔΔCt between FTP and LTP of patients. * denotes a p-value≤0.05, ** denotes a p-value≤0.01 and *** denotes a p-value≤0.001.


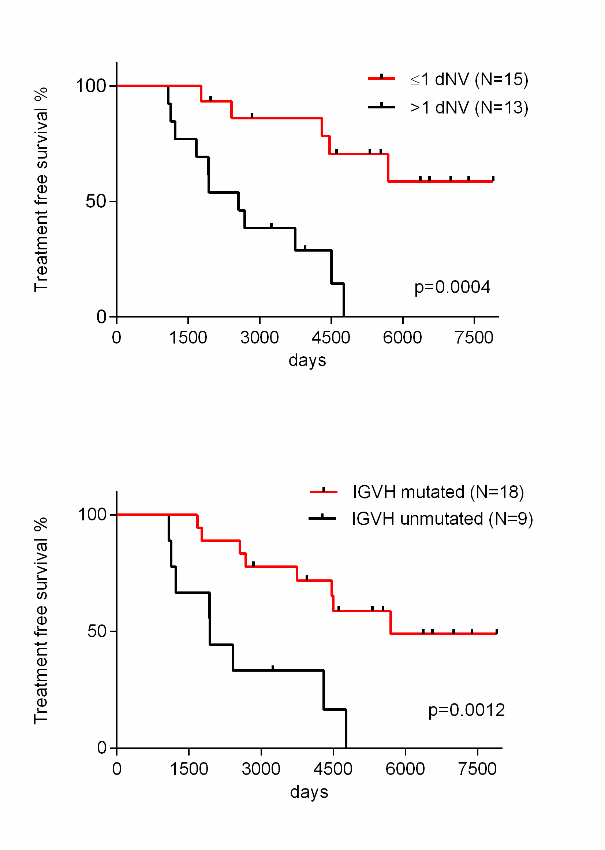


**Fig.S6.** Kaplan-Meier curve of treatment-free survival in CLL patients dichotomized based on the number of dNVs (NVs that change more than 20% between FTP and LTP) (upper panel) and on the mutational status of the poor prognostic factor IGVH (lower panel). The median of the dNVs number across all the samples was used as cut-off. Time to treatment was calculated from the diagnosis; the last follow-up was considered for patients which did not undergo to treatment. The Log-rank test was used.


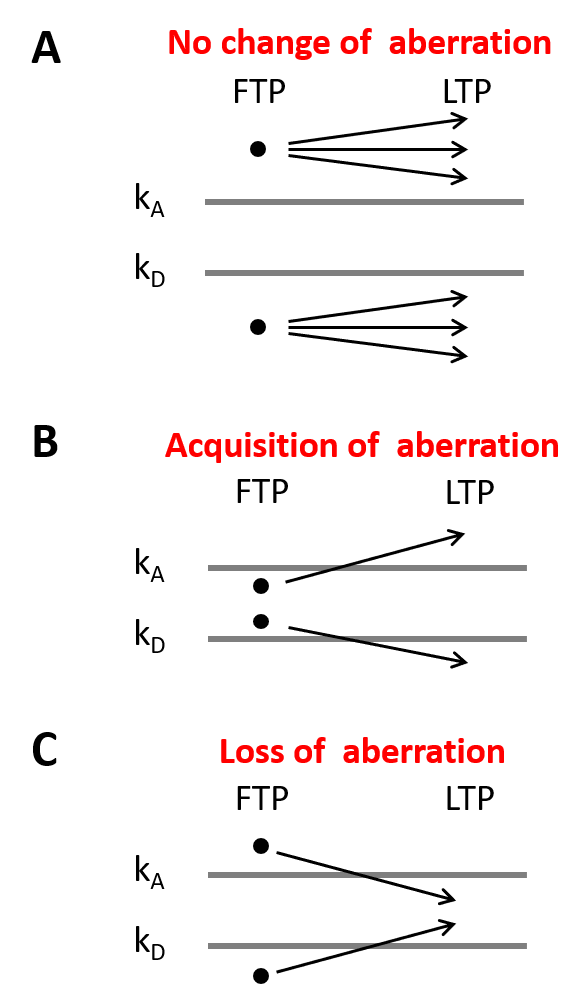


Fig.S7: Classes assignment of the PS, based on their *k* at the FTP and LTP. A segment whose *k* value exceeds either the k_A_ or the k_D_ thresholds (for an amplification or deletion, respectively) has been considered as aberrant. A segment, which is aberrant in at least one of the two temporal acquisitions, has been assigned to one of these three classes: A) No change of aberration: the segment is aberrant in both FTP and LTP; B) Acquisition of aberration: the segment is aberrant only in the LTP; C) Loss of aberration: the segment is aberrant only in the LTP.


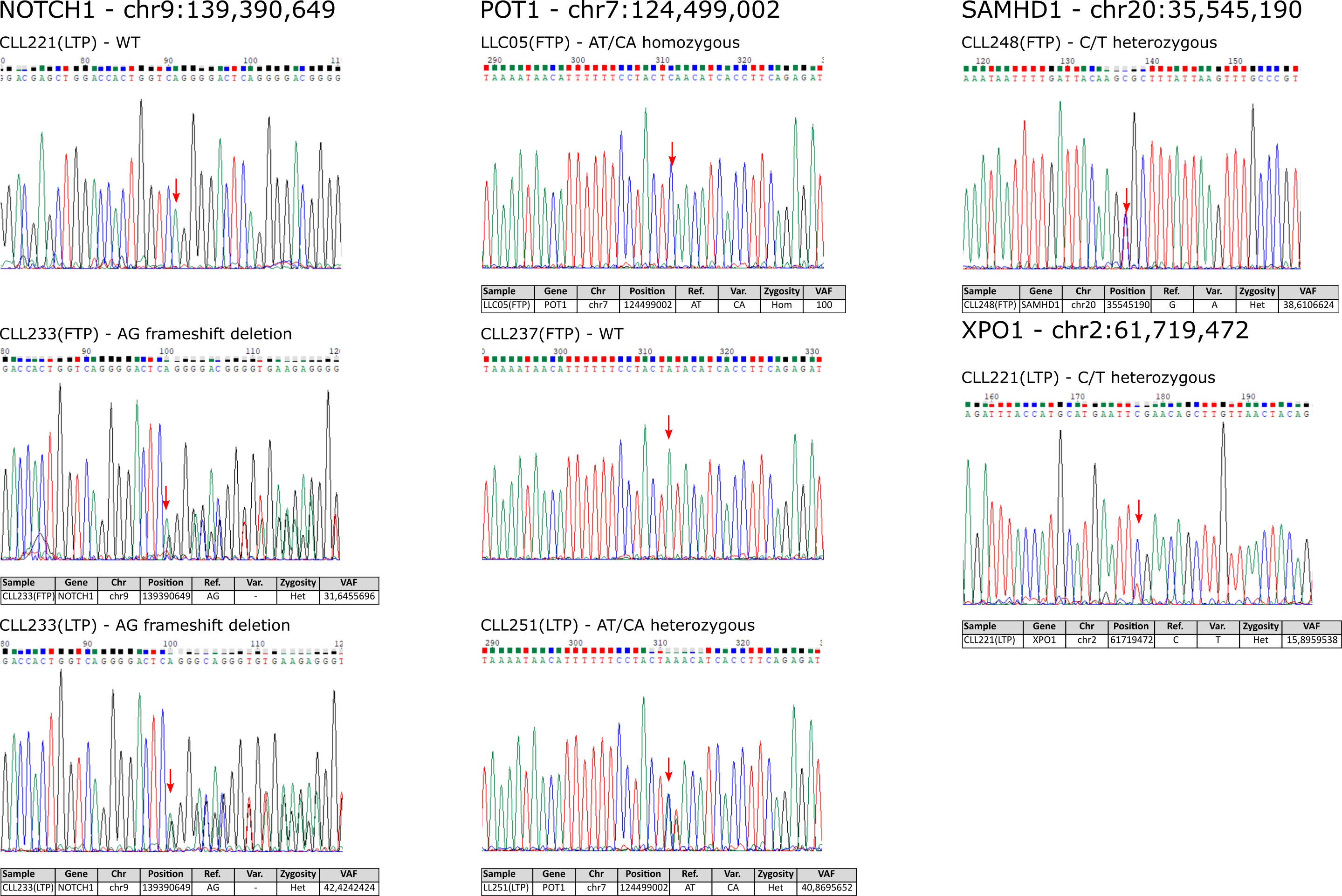


**Fig.S8: Sanger sequencing validation of some of the mutation observed by NGS.** A frameshift deletion in NOTCH1 gene, a dinucleotide variation in POT1 gene, a single nucleotide variation in SAMHD1 and XPO1 genes identified by NGS were analysed by sanger sequencing. For each patient showing the mutation, Sanger chromatogram (upper) and NGS results (lower) are shown.





**Fig.S9:** **Longitudinal analysis of copy number variations for chromosomal regions carrying CLL-deregulated miRNAs.** LogR values of selected miRNAs were obtained after Rawcopy processing of SNP array data. These LogR values at the FTP and LTP of 11 S-CLL and 17 P-CLL were plotted in box and whisker graph showing median and min to max. Unpaired test was used to compare the FTP and the LTP between groups (FTP-P-CLL*vs*FTP-S-CLL and LTP-P-CLLvsLTP-S-CLL). Paired test was used to compare the FTP and LTP of S-CLL and P-CLL; parametric or not parametric tests were used according to normality test. Statistical tests were two-sided, * denotes a p-value≤0.05, ** denotes a p-value≤0.01 and *** denotes a p-value≤0.001.

**Table S5.** Oligonucleotide sequences used for CNV analysis by qPCR (CNV) and conventional Sanger sequencing (PCR-Seq). The UPLs-Locked Nucleic Acids (Universal Probe library, Roche) probes used for CNV analysis by qPCR are indicated; Fam is the 6-carboxyfluorescein dye whereas Q is the fluorescein quencher.

| ***Gene*** | **Name** | **Sequence F** | **Sequence R** | **Probe (Sequence)** | **Methodology** |
| --- | --- | --- | --- | --- | --- |
| *ATM* | U7_ATM | TTGTATCATGGATGTGTCATTACG | GCTGTCTGGCAAACCTGACT | UPL7  (Fam- -Q) | qPCR (CNV) |
| *TP53* | U5_TP53 | TGTTCTTGCAGTTAAGGGTTAGTTT | TGAAGTGGGCCCCTACCTA | UPL5  (Fam- -Q) | qPCR (CNV) |
| *ASUN* | U27_ASUN | GTGTTTGTTGTGGATCACTGC | CTTCACCAGCATATCAAACTCG | UPL27  (Fam- -Q) | qPCR (CNV) |
| *HAL* | U51_HAL | GAAAGTGGACAGGAGGCTCA | TGGTTTTTGTAGCCGAGCA | UPL51  (Fam- -Q) | qPCR (CNV) |
| *NOTCH1* | NOTCH1 | ACTTGAAGGCCTCCGGAATG | TGCACACTATTCTGCCCCAG |  | PCR-Seq |
| *POT1* | POT1 | ACCATGTTCATGTGGCAAGA | CCAGTTTACCAAGCTTAGCATTT |  | PCR-Seq |
| *SAMHD1* | SAMHD1 | TCACAGACACGGGCAAACTT | TGCCAGGTATGCACTGAACA |  | PCR-Seq |
| *XPO1* | XPO1 | CTGCGGCATTTTTGGGCTAT | AGCAATGCATGAAGAGGACGA |  | PCR-Seq |

1. Hallek M, Cheson BD, Catovsky D, Caligaris-Cappio F, Dighiero G, Dohner H, et al. Guidelines for the diagnosis and treatment of chronic lymphocytic leukemia: a report from the International Workshop on Chronic Lymphocytic Leukemia updating the National Cancer Institute-Working Group 1996 guidelines. Blood. 2008;111(12):5446-56.

2. Mayrhofer M, Viklund B, Isaksson A. Rawcopy: Improved copy number analysis with Affymetrix arrays. Sci Rep. 2016;6:36158.

3. Van Loo P, Nordgard SH, Lingjaerde OC, Russnes HG, Rye IH, Sun W, et al. Allele-specific copy number analysis of tumors. Proc Natl Acad Sci U S A. 2010;107(39):16910-5.

4. Landau Dan A, Carter Scott L, Stojanov P, McKenna A, Stevenson K, Lawrence Michael S, et al. Evolution and Impact of Subclonal Mutations in Chronic Lymphocytic Leukemia. Cell. 2013;152(4):714-26.

5. Carter SL, Cibulskis K, Helman E, McKenna A, Shen H, Zack T, et al. Absolute quantification of somatic DNA alterations in human cancer. Nat Biotechnol. 2012;30(5):413-21.

6. Puente XS, Pinyol M, Quesada V, Conde L, Ordóñez GR, Villamor N, et al. Whole-genome sequencing identifies recurrent mutations in chronic lymphocytic leukaemia. Nature. 2011;475(7354):101-5.

7. Quesada V, Conde L, Villamor N, Ordonez GR, Jares P, Bassaganyas L, et al. Exome sequencing identifies recurrent mutations of the splicing factor SF3B1 gene in chronic lymphocytic leukemia. Nat Genet. 2011;44(1):47-52.

8. Wang L, Lawrence MS, Wan Y, Stojanov P, Sougnez C, Stevenson K, et al. SF3B1and Other Novel Cancer Genes in Chronic Lymphocytic Leukemia. New England Journal of Medicine. 2011;365(26):2497-506.

9. Rossi D, Fangazio M, Rasi S, Vaisitti T, Monti S, Cresta S, et al. Disruption of BIRC3 associates with fludarabine chemorefractoriness in TP53 wild-type chronic lymphocytic leukemia. Blood. 2012;119(12):2854-62.

10. Jebaraj BM, Kienle D, Buhler A, Winkler D, Dohner H, Stilgenbauer S, et al. BRAF mutations in chronic lymphocytic leukemia. Leuk Lymphoma. 2013;54(6):1177-82.

11. Marincevic M, Tobin G, Rosenquist R. Infrequent occurrence of PIK3CA mutations in chronic lymphocytic leukemia. Leuk Lymphoma. 2009;50(5):829-30.

12. Lupini L, Bassi C, Mlcochova J, Musa G, Russo M, Vychytilova-Faltejskova P, et al. Prediction of response to anti-EGFR antibody-based therapies by multigene sequencing in colorectal cancer patients. BMC Cancer. 2015;15(1).

13. Chiorazzi N. Implications of new prognostic markers in chronic lymphocytic leukemia. Hematology Am Soc Hematol Educ Program. 2012;2012:76-87.

14. Fulci V, Chiaretti S, Goldoni M, Azzalin G, Carucci N, Tavolaro S, et al. Quantitative technologies establish a novel microRNA profile of chronic lymphocytic leukemia. Blood. 2007;109(11):4944-51.

15. Visone R, Veronese A, Balatti V, Croce CM. MiR-181b: new perspective to evaluate disease progression in chronic lymphocytic leukemia. Oncotarget. 2012;3(2):195-202.

16. Balatti V, Pekarky Y, Croce CM. Role of microRNA in chronic lymphocytic leukemia onset and progression. J Hematol Oncol. 2015;8:12.
