# Supplementary material for: Genetic dynamics in untreated CLL patients with either stable or progressive disease: a longitudinal study
Source: J Hematol Oncol. 2019 Nov 19;12:114. doi: 10.1186/s13045-019-0802-x (PMC6862808; doi:10.1186/s13045-019-0802-x)
Supplement: Supplementary file 3 — Additional file 3: Table S2 HaloPlex SureDesign Report. [file 13045_2019_802_MOESM3_ESM.docx]

**Table S2.** **HaloPlex SureDesign Report.** Libraries of regions were designed by SureDesign Custom Design Tool. Target ID, the unique code assigned to a target by Agilent; interval, the genomic interval of a target; gene name, gene within the target; regions size, amplicons within a target.

| **Gene name** | **TargetID** | **Interval** | **Regions Size** | **Databases** | **References** |
| --- | --- | --- | --- | --- | --- |
| **EGR2** | 1959 | chr10:64572956-64578409 | 1590 | CCDS, Ensembl, Gencode, RefSeq, VEGA | [1] |
| **POT1** | 25913 | chr7:124462571-124540896 | 2450 | CCDS, Ensembl, Gencode, RefSeq, VEGA | [1, 2] |
| **MYD88** | 4615 | chr3:38180142-38182787 | 1054 | CCDS, Ensembl, Gencode, RefSeq, VEGA | [1, 3, 4] |
| **ATM** | 472 | chr11:108098341-108236245 | 10411 | CCDS, Ensembl, Gencode, RefSeq | [1, 4] |
| **BCOR** | 54880 | chrX:39909158-39937192 | 5707 | CCDS, Ensembl, Gencode, RefSeq, VEGA | [1] |
| **FBXW7** | 55294 | chr4:153244022-153332965 | 2898 | CCDS, Ensembl, Gencode, RefSeq | [4] |
| **TP53** | 7157 | chr17:7565246-7579922 | 1697 | CCDS, Ensembl, Gencode, RefSeq, VEGA | [1, 4] |
| **DDX3Y** | 8653 | chrY:15016837-15030044 | 2379 | CCDS, Ensembl, Gencode, RefSeq, VEGA | COSMIC |
| **BIRC3** | chr11:102190608-102190737 | chr11:102190608-102190737 | 130 | CustomRegion | [5] |
|  | chr11:102192568-102196093 | chr11:102192568-102196093 | 3526 | CustomRegion |  |
|  | chr11:102201721-102201974 | chr11:102201721-102201974 | 254 | CustomRegion |  |
|  | chr11:102207590-102207753 | chr11:102207590-102207753 | 164 | CustomRegion |  |
|  | chr11:102207640-102208465 | chr11:102207640-102208465 | 826 | CustomRegion |  |
| **KRAS** | chr12:25398208-25398329 | chr12:25398208-25398329 | 122 | CustomRegion | [1] |
|  | chr12:25403685-25403865 | chr12:25403685-25403865 | 181 | CustomRegion |  |
| **CHD2** | chr15:93498653-93498742 | chr15:93498653-93498742 | 90 | CustomRegion | [1, 2, 6-8] COSMIC |
|  | chr15:93499689-93499879 | chr15:93499689-93499879 | 191 | CustomRegion |  |
|  | chr15:93510555-93510743 | chr15:93510555-93510743 | 189 | CustomRegion |  |
|  | chr15:93514995-93515157 | chr15:93514995-93515157 | 163 | CustomRegion |  |
|  | chr15:93515495-93515647 | chr15:93515495-93515647 | 153 | CustomRegion |  |
|  | chr15:93518109-93518180 | chr15:93518109-93518180 | 72 | CustomRegion |  |
|  | chr15:93521464-93521613 | chr15:93521464-93521613 | 150 | CustomRegion |  |
|  | chr15:93522365-93522513 | chr15:93522365-93522513 | 149 | CustomRegion |  |
|  | chr15:93524045-93524141 | chr15:93524045-93524141 | 97 | CustomRegion |  |
|  | chr15:93524595-93524687 | chr15:93524595-93524687 | 93 | CustomRegion |  |
|  | chr15:93527560-93527730 | chr15:93527560-93527730 | 171 | CustomRegion |  |
|  | chr15:93528728-93528903 | chr15:93528728-93528903 | 176 | CustomRegion |  |
|  | chr15:93534706-93534747 | chr15:93534706-93534747 | 42 | CustomRegion |  |
|  | chr15:93536089-93536228 | chr15:93536089-93536228 | 140 | CustomRegion |  |
|  | chr15:93540187-93540325 | chr15:93540187-93540325 | 139 | CustomRegion |  |
|  | chr15:93540483-93540633 | chr15:93540483-93540633 | 151 | CustomRegion |  |
|  | chr15:93541729-93541851 | chr15:93541729-93541851 | 123 | CustomRegion |  |
|  | chr15:93543742-93543870 | chr15:93543742-93543870 | 129 | CustomRegion |  |
|  | chr15:93545407-93545547 | chr15:93545407-93545547 | 141 | CustomRegion |  |
|  | chr15:93547847-93547981 | chr15:93547847-93547981 | 135 | CustomRegion |  |
|  | chr15:93552375-93552553 | chr15:93552375-93552553 | 179 | CustomRegion |  |
|  | chr15:93555575-93555674 | chr15:93555575-93555674 | 100 | CustomRegion |  |
| **TP53** | chr17:7576910-7577181 | chr17:7576910-7577181 | 272 | CustomRegion | [1, 4] |
|  | chr17:7577489-7577620 | chr17:7577489-7577620 | 132 | CustomRegion |  |
|  | chr17:7578121-7578320 | chr17:7578121-7578320 | 200 | CustomRegion |  |
|  | chr17:7578360-7578622 | chr17:7578360-7578622 | 263 | CustomRegion |  |
|  | chr17:7579258-7579621 | chr17:7579258-7579621 | 364 | CustomRegion |  |
| **NRAS** | chr1:115256462-115256609 | chr1:115256462-115256609 | 148 | CustomRegion | [1] |
|  | chr1:115258622-115258794 | chr1:115258622-115258794 | 173 | CustomRegion |  |
|  | chr1:115259279-115259515 | chr1:115259279-115259515 | 237 | CustomRegion |  |
| **ITPKB** | chr1:226923228-226925364 | chr1:226923228-226925364 | 2137 | CustomRegion | [1] |
| **SAMHD1** | chr20:35545125-35545233 | chr20:35545125-35545233 | 109 | CustomRegion | [1, 7, 9] |
|  | chr20:35545352-35545452 | chr20:35545352-35545452 | 101 | CustomRegion |  |
|  | chr20:35547767-35547922 | chr20:35547767-35547922 | 156 | CustomRegion |  |
|  | chr20:35555585-35555655 | chr20:35555585-35555655 | 71 | CustomRegion |  |
|  | chr20:35559163-35559278 | chr20:35559163-35559278 | 116 | CustomRegion |  |
| **MAPK1** | chr22:22127105-22127367 | chr22:22127105-22127367 | 263 | CustomRegion | [1] |
|  | chr22:22142546-22142677 | chr22:22142546-22142677 | 132 | CustomRegion |  |
|  | chr22:22142983-22143097 | chr22:22142983-22143097 | 115 | CustomRegion |  |
|  | chr22:22160113-22160242 | chr22:22160113-22160242 | 130 | CustomRegion |  |
|  | chr22:22161953-22162135 | chr22:22161953-22162135 | 183 | CustomRegion |  |
| **SF3B1** | chr2:198265439-198265660 | chr2:198265439-198265660 | 222 | CustomRegion | [1, 2, 4] |
|  | chr2:198266466-198266612 | chr2:198266466-198266612 | 147 | CustomRegion |  |
|  | chr2:198266709-198266854 | chr2:198266709-198266854 | 146 | CustomRegion |  |
|  | chr2:198267280-198267550 | chr2:198267280-198267550 | 271 | CustomRegion |  |
| **XPO1** | chr2:61719460-61719616 | chr2:61719460-61719616 | 157 | CustomRegion | [1, 3] |
|  | chr2:61722590-61722748 | chr2:61722590-61722748 | 159 | CustomRegion |  |
| **KLHL6** | chr3:183273149-183273477 | chr3:183273149-183273477 | 329 | CustomRegion | [3] |
| **HIST1H1E** | chr6:26156608-26157288 | chr6:26156608-26157288 | 681 | CustomRegion | [1] |
| **RIPK1** | chr6:3083319-3083547 | chr6:3083319-3083547 | 229 | CustomRegion | [1, 10] |
|  | chr6:3085493-3085642 | chr6:3085493-3085642 | 150 | CustomRegion |  |
|  | chr6:3089815-3089891 | chr6:3089815-3089891 | 77 | CustomRegion |  |
|  | chr6:3104459-3104549 | chr6:3104459-3104549 | 91 | CustomRegion |  |
|  | chr6:3105716-3106285 | chr6:3105716-3106285 | 570 | CustomRegion |  |
|  | chr6:3111037-3111189 | chr6:3111037-3111189 | 153 | CustomRegion |  |
| **BRAF** | chr7:140453075-140453193 | chr7:140453075-140453193 | 119 | CustomRegion | [11] |
|  | chr7:140453086-140453260 | chr7:140453086-140453260 | 175 | CustomRegion |  |
| **NOTCH1** | chr9:139388896-139392010 | chr9:139388896-139392010 | 3115 | CustomRegion | [2-4, 12] |
| **DDX3X** | chrx:41192651-41194630 | chrx:41192651-41194630 | 1980 | CustomRegion | [1, 4, 10] |
|  | chrx:41196641-41196765 | chrx:41196641-41196765 | 125 | CustomRegion |  |
|  | chrx:41204359-41204528 | chrx:41204359-41204528 | 170 | CustomRegion |  |
|  | chrx:41204659-41204807 | chrx:41204659-41204807 | 149 | CustomRegion |  |
| **MED12** | chrx:70338406-70338703 | chrx:70338406-70338703 | 298 | CustomRegion | [1] |
| **ZMYM3** | chrx:70461077-70461194 | chrx:70461077-70461194 | 118 | CustomRegion | [1, 4, 8] COSMIC |
|  | chrx:70462020-70462274 | chrx:70462020-70462274 | 255 | CustomRegion |  |
|  | chrx:70462820-70462934 | chrx:70462820-70462934 | 115 | CustomRegion |  |
|  | chrx:70463679-70463830 | chrx:70463679-70463830 | 152 | CustomRegion |  |
|  | chrx:70464152-70464320 | chrx:70464152-70464320 | 169 | CustomRegion |  |
|  | chrx:70464640-70464743 | chrx:70464640-70464743 | 104 | CustomRegion |  |
|  | chrx:70465189-70465335 | chrx:70465189-70465335 | 147 | CustomRegion |  |
|  | chrx:70465518-70465692 | chrx:70465518-70465692 | 175 | CustomRegion |  |
|  | chrx:70465836-70465948 | chrx:70465836-70465948 | 113 | CustomRegion |  |
|  | chrx:70469876-70470053 | chrx:70469876-70470053 | 178 | CustomRegion |  |
|  | chrx:70472439-70473124 | chrx:70472439-70473124 | 686 | CustomRegion |  |
|  | chrx:70474318-70474996 | chrx:70474318-70474996 | 679 | CustomRegion |  |
| **KIT** | chr4:55561678-55561947 | chr4:55561678-55561947 | 270 | CustomRegion | [12] |
|  | chr4:55594177-55594287 | chr4:55594177-55594287 | 111 | CustomRegion |  |
|  | chr4:55595501-55595651 | chr4:55595501-55595651 | 151 | CustomRegion |  |
|  | chr4:55595502-55595653 | chr4:55595502-55595653 | 152 | CustomRegion |  |
|  | chr4:55602602-55602776 | chr4:55602602-55602776 | 175 | CustomRegion |  |
| **PIK3CA** | chr3:178916538-178916965 | chr3:178916538-178916965 | 428 | CustomRegion | [13] |
|  | chr3:178951952-178952125 | chr3:178951952-178952125 | 174 | CustomRegion |  |

**References**:

1. Landau Dan A, Carter Scott L, Stojanov P, McKenna A, Stevenson K, Lawrence Michael S, Sougnez C, Stewart C, Sivachenko A, Wang L *et al*: **Evolution and Impact of Subclonal Mutations in Chronic Lymphocytic Leukemia**. *Cell* 2013, **152**(4):714-726.

2. Quesada V, Conde L, Villamor N, Ordonez GR, Jares P, Bassaganyas L, Ramsay AJ, Bea S, Pinyol M, Martinez-Trillos A *et al*: **Exome sequencing identifies recurrent mutations of the splicing factor SF3B1 gene in chronic lymphocytic leukemia**. *Nat Genet* 2011, **44**(1):47-52.

3. Puente XS, Pinyol M, Quesada V, Conde L, Ordóñez GR, Villamor N, Escaramis G, Jares P, Beà S, González-Díaz M *et al*: **Whole-genome sequencing identifies recurrent mutations in chronic lymphocytic leukaemia**. *Nature* 2011, **475**(7354):101-105.

4. Wang L, Lawrence MS, Wan Y, Stojanov P, Sougnez C, Stevenson K, Werner L, Sivachenko A, DeLuca DS, Zhang L *et al*: **SF3B1and Other Novel Cancer Genes in Chronic Lymphocytic Leukemia**. *New England Journal of Medicine* 2011, **365**(26):2497-2506.

5. Rossi D, Fangazio M, Rasi S, Vaisitti T, Monti S, Cresta S, Chiaretti S, Del Giudice I, Fabbri G, Bruscaggin A *et al*: **Disruption of BIRC3 associates with fludarabine chemorefractoriness in TP53 wild-type chronic lymphocytic leukemia**. *Blood* 2012, **119**(12):2854-2862.

6. Ljungstrom V, Cortese D, Young E, Pandzic T, Mansouri L, Plevova K, Ntoufa S, Baliakas P, Clifford R, Sutton LA *et al*: **Whole-exome sequencing in relapsing chronic lymphocytic leukemia: clinical impact of recurrent RPS15 mutations**. *Blood* 2016, **127**(8):1007-1016.

7. Roberts KG, Li Y, Payne-Turner D, Harvey RC, Yang YL, Pei D, McCastlain K, Ding L, Lu C, Song G *et al*: **Targetable kinase-activating lesions in Ph-like acute lymphoblastic leukemia**. *N Engl J Med* 2014, **371**(11):1005-1015.

8. Ma X, Edmonson M, Yergeau D, Muzny DM, Hampton OA, Rusch M, Song G, Easton J, Harvey RC, Wheeler DA *et al*: **Rise and fall of subclones from diagnosis to relapse in pediatric B-acute lymphoblastic leukaemia**. *Nat Commun* 2015, **6**:6604.

9. Messina M, Del Giudice I, Khiabanian H, Rossi D, Chiaretti S, Rasi S, Spina V, Holmes AB, Marinelli M, Fabbri G *et al*: **Genetic lesions associated with chronic lymphocytic leukemia chemo-refractoriness**. *Blood* 2014, **123**(15):2378-2388.

10. Gunawardana J, Chan FC, Telenius A, Woolcock B, Kridel R, Tan KL, Ben-Neriah S, Mottok A, Lim RS, Boyle M *et al*: **Recurrent somatic mutations of PTPN1 in primary mediastinal B cell lymphoma and Hodgkin lymphoma**. *Nat Genet* 2014, **46**(4):329-335.

11. Jebaraj BM, Kienle D, Buhler A, Winkler D, Dohner H, Stilgenbauer S, Zenz T: **BRAF mutations in chronic lymphocytic leukemia**. *Leuk Lymphoma* 2013, **54**(6):1177-1182.

12. Landau DA, Carter SL, Stojanov P, McKenna A, Stevenson K, Lawrence MS, Sougnez C, Stewart C, Sivachenko A, Wang L *et al*: **Evolution and impact of subclonal mutations in chronic lymphocytic leukemia**. *Cell* 2013, **152**(4):714-726.

13. Marincevic M, Tobin G, Rosenquist R: **Infrequent occurrence of PIK3CA mutations in chronic lymphocytic leukemia**. *Leuk Lymphoma* 2009, **50**(5):829-830.
