# Supplementary material for: Genetic dynamics in untreated CLL patients with either stable or progressive disease: a longitudinal study
Source: J Hematol Oncol. 2019 Nov 19;12:114. doi: 10.1186/s13045-019-0802-x (PMC6862808; doi:10.1186/s13045-019-0802-x)
Supplement: Supplementary file 4 — Additional file 4: Table S3 Genetic variants represented in Fig. 2. [file 13045_2019_802_MOESM4_ESM.pdf]

	A	B	C	D	E	F	G	H	I	J	K	L	M	N	O	P	Q	R	S	T
1	**Supplemental Table 3.** Genetic variants represented in Figure 2.																			
2	**Stable**											**VAF**			**Qual***		**Coverage***			
3		**Location**	**Gene**	**Chr**	**Position**	**Ref.**	**Var.**	**Zygosity**	**dbSNP ID**	**MAF (1000 genomes)**	**Mutation Type**	**FTP**	**LTP**	**N° of mutation**	**FTP**	**LTP**	**FTP**	**LTP**	**PP2 Index***	**AAChange***
4	**CLL478**	exonic	ATM	chr11	108124610	T	-	Het.			**frameshift deletion**		18,4	2		56,17		179		NM_000051: c.1968delT:p.T656fs
5		UTR3	NOTCH1	chr9	139389729	C	T	Het.				22,7	31,6		360,04	675,84	295	316		
6	**CLL504**	exonic	MYD88	chr3	38181367	A	-	Het.			**frameshift deletion**	11,2	17,1	1	47,15	142,57	1975	1582		NM_001172567: c.380delA:p.Q127fs
7	**CLL514**	exonic	MYD88	chr3	38181367	A	-	Het.			**frameshift deletion**	20,8	15,1	1	286,66	91,72	1493	1241		NM_001172567: c.380delA:p.Q127fs
8	**CLL534**	exonic	CHD2	chr15	93527650	A	-	Het.			**frameshift deletion**	20,3		1	152,3		577			NM_001271: c.3157delA:p.K1053fs
9	**CLL538**	exonic	MYD88	chr3	38181367	A	-	Het.			**frameshift deletion**	17,5		2	113,48		988			NM_001172567: c.380delA:p.Q127fs
10		exonic	ATM	chr11	108124610	T	-	Het.			**frameshift deletion**	19,2			45,98		172			NM_000051: c.1968delT:p.T656fs
11	**LLC003**	exonic	ITPKB	chr1	226924628	A	G	Het			**nonsynonymous SNV**	8,661417		1	52,46		254		0,966	NM_002221: c.T532C:p.C178R
12	**LLC005**	exonic	ITPKB	chr1	226923871	G	-	Het			**frameshift deletion**	70,87379		1	100		103			NM_002221: c.1289delC:p.A430fs
13	**LLC008**	intronic	POT1	chr7	124499003	T	-	Het.	rs201732238				38,2	2		348,05		110		
14		exonic	ITPKB	chr1	226923497	G	A	Het.			**nonsynonymous SNV**	14	11		261,81	305,15	430	809	0,666	NM_002221: c.C1663T:p.P555S
15	**LLC014**	exonic	ITPKB	chr1	226924628	A	G	Het			**nonsynonymous SNV**	9,090909		2	100		517		0,966	NM_002221: c.T532C:p.C178R
16		UTR3	BIRC3	chr11	102207851	G	A	Het	rs1055088	G=0.3437/1721		60,30534	44,62366		100	100	131	186		
17	**LLC035**													0						
18	**LLC052**													0						
19														1,181818182						
20	**Progressive**											**VAF**			**Qual***		**Coverage***			
21		**Location**	**Gene**	**Chr**	**Position**	**Ref.**	**Var.**	**Zygosity**	**dbSNP ID**	**MAF(1000 genomes)**	**Mutation Type**	**FTP**	**LTP**	**N° of mutation**	**FTP**	**LTP**	**FTP**	**LTP**	**PP2 Index***	**AAChange***
22	**CLL200**	exonic	ITPKB	chr1	226924628	A	G	Het			**nonsynonymous SNV**	9,401709		1	51,87		234		0,966	NM_002221: c.T532C:p.C178R
23	**CLL206**													0						
24	**CLL211**	exonic	PIK3CA	chr3	178916793	A	G	Het			**synonymous SNV**		10,54852	2		100		474		NM_006218: c.A180G:p.Q60Q
25		UTR3	NOTCH1	chr9	139389184	A	G	Het	rs6563	A=0.4954/2481		36,00973	46,30021		100	100	411	473		
26	**CLL221**	exonic	ITPKB	chr1	226923871	G	C	Het			**nonsynonymous SNV**	57,97101		3	100		138		0,001	NM_002221: c.C1289G:p.A430G
27		exonic	XPO1	chr2	61719472	C	T	Het			**nonsynonymous SNV**	6,382979	15,9		62,84	100	517	346	0,999	NM_003400: c.G1711A:p.E571K
28		exonic	NOTCH1	chr9	139390732	GCG	-	Het			**nonframeshift deletion**		14,73566			100		889		NM_017617: c.7457_7459del:p.2486_2487del
29	**CLL227**	exonic	TP53	chr17	7577556	C	G	Het			**nonsynonymous SNV**	40,88398	83,5249	2	100	100	362	261	1	NM_001126115: c.G329C:p.C110S
30		exonic	ITPKB	chr1	226924573	CGGGCGC	CGCGC	Het			**frameshift substitution**	96,59091			100		176			ITPKB:NM_002221:exon2: c.581_587GCGCG
31	**CLL233**	exonic	EGR2	chr10	64573248	G	T	Het			**nonsynonymous SNV**	35,7	44,75	2	100	100	2000	2000	0,977	NM_000399: c.C1150A:p.H384N
32		exonic	NOTCH1	chr9	139390649	AG	-	Het			**frameshift deletion**	31,64557	42,42424		100	100	158	132		NM_017617: c.7541_7542del:p.2514_2514del
33	**CLL237**													0						
34	**CLL242**	exonic	ATM	chr11	108188136	G	A	Het	rs1800060	A=0.0028/14	**nonsynonymous SNV**	41,46341	50,72993	5	100	100	205	274	0	NM_000051: c.G6235A:p.V2079I
35		exonic	CHD2	chr15	93521567	A	T	Het			**nonsynonymous SNV**	3,541913	5,464481		16,33	44,9	847	549	0,999	NM_001271: c.A2681T:p.D894V
36		exonic	ITPKB	chr1	226923264	G	A	Het	rs708775	A=0.2815/1410	**synonymous SNV**	49,21875			100		128			NM_002221: c.C1896T:p.A632A
37		exonic	ZMYM3	chrX	70465279	G	C	Het			**nonsynonymous SNV**	11,27451			90,41		204		0,981	NM_001171162: c.C2881G:p.P961A
38		UTR3	BIRC3	chr11	102208390	G	C	Het	rs3460	C=0.0891/446		43,54839	55,02959		100	100	124	169		
39	**CLL248**	exonic	SAMHD1	chr20	35545190	G	A	Het			**nonsynonymous SNV**	38,61066	46,82203	3	100	100	619	472	1	NM_015474: c.C997T:p.R333C
40		exonic	ITPKB	chr1	226923871	G	-	Het			**frameshift deletion**	97,05882			100		102			NM_002221: c.1289delC:p.A430fs
41		UTR5	ITPKB	chr1	226925167	G	A	Het	rs138545670				19,54887			100		133		
42	**CLL251**	exonic	ITPKB	chr1	226924630	G	-	Het			**frameshift deletion**		72,0339	2		100		118		NM_002221: c.530delC:p.P177fs
43		intronic	POT1	chr7	124499002	AT	CA	Het					40,86957			100		115		
44	**CLL259**													0						
45	**CLL262a**	exonic	ITPKB	chr1	226923264	G	A	Het	rs708775	A=0.2815/1410	**synonymous SNV**		45,31646	11		100		395		NM_002221: c.C1896T:p.A632A
46		exonic	ITPKB	chr1	226923505	G	T	Het	rs708776	G=0.0202/101	**nonsynonymous SNV**	39,1129	100		100	100	248	340	0	NM_002221: c.C1655A:p.P552Q
47		exonic	ITPKB	chr1	226923938	A	C	Het	rs6667260	A=0.3896/1951	**nonsynonymous SNV**	42,29249	100		100	100	253	383	0	NM_002221: c.T1222G:p.S408A
48		exonic	ATM	chr11	108160480	T	G	Het	rs138327406	G=0.0004/2	**nonsynonymous SNV**	37,86408			100		103		0,999	NM_000051: c.T4388G:p.F1463C
49		exonic	MAPK1	chr22	22162126	A	G	Het	rs3729910	G=0.0306/153	**synonymous SNV**	59,95976			100		497			NM_002745: c.T129C:p.Y43Y
50		exonic	NOTCH1	chr9	139391636	G	A	Hom	rs2229974	G=0.3049/1527	**synonymous SNV**	100			100		470			NM_017617: c.C6555T:p.D2185D
51		exonic	BCOR	chrX	39932907	T	C	Het	rs6520618	T=0.4760/1797	**synonymous SNV**	39,27928			100		555			NM_001123383: c.A1692G:p.A564A
52		intronic	POT1	chr7	124540872	C	T	Hom	rs7782354	C=0.4185/2096		100			100		126			
53		intronic	DDX3X	chrX	41193745	C	T	Het	rs184347580	T=0.0543/205		34,64567			100		127			
54		UTR5	MED12	chrX	70338420	C	T	Het				51,55807			100		353			
55		UTR5	BIRC3	chr11	102192744	T	C	Het	rs17880156	C=0.0070/35			51,33333			100		300		
56	**CLL400t**													0						
57	**CLL408**	UTR3	NOTCH1	chr9	139389184	A	G	Hom	rs6563	A=0.4954/2481		99,08257		1	100		109			
58	**CLL416**													0						
59	**CLL523**	exonic	NOTCH1	chr9	139390649	AG	-	Het			**frameshift deletion**		16,47597	1		100		437		NM_017617: c.7541_7542del:p.2514_2514del
60	**LLC034**	exonic	MYD88	chr3	38181367	A	-	Het.			**frameshift deletion**		17,4	24	191,85		1990			NM_001172567: c.380delA:p.Q127fs
61		exonic	ITPKB	chr1	226923264	G	A	Het.	rs708775	A=0.2815/1410	**synonymous SNV**	44,1	94,5		6864,04	15261,9	1690	1101		NM_002221: c.C1896T:p.A632A
62		exonic	ITPKB	chr1	226923504	-	T	Hom.			**frameshift insertion**	100			15652,5		1190			NM_002221: c.1655_1656insA:p.P552fs
63		exonic	ITPKB	chr1	226923507	A	-	Het.			**frameshift deletion**	11,1			458,5		1195			NM_002221: c.1653delT:p.D551fs
64		exonic	ITPKB	chr1	226923497	G	A	Het.			**nonsynonymous SNV**	11,4			477,22		1193		0,666	NM_002221: c.C1663T:p.P555S
65		exonic	ITPKB	chr1	226923938	A	C	Het.	rs6667260	A=0.3896/1951	**nonsynonymous SNV**	52,2	100		6529,36	11821	1199	752	0	NM_002221: c.T1222G:p.S408A
66		exonic	ITPKB	chr1	226924991	G	A	Het.			**nonsynonymous SNV**		56,5			1066,53		177	0,983	NM_002221: c.C169T:p.L57F
67		intronic	ATM	chr11	108160576	T	G	Het.					43,4			2228,92		555		
68		exonic	ATM	chr11	108175462	G	A	Het.	rs1801516	A=0.0669/335	**nonsynonymous SNV**		40			370,55		100	0,044	NM_000051: c.G5557A:p.D1853N
69		exonic	CHD2	chr15	93536197	C	T	Het.	rs2272457	T=0.1935/969	**synonymous SNV**	46,6			8073,03		1778			NM_001271: c.C3564T:p.Y1188Y
70		exonic	CHD2	chr15	93545488	T	A	Het.	rs61756301	A=0.0088/44	**nonsynonymous SNV**	63,8			11598,1		1254		0,187	NM_001271: c.T4219A:p.S1407T
71		exonic	NRAS	chr1	115256529	T	C	Het.	rs11554290		**nonsynonymous SNV**		28,8		2854,54		1363		0,214	NM_002524: c.A182G:p.Q61R
72		exonic	SF3B1	chr2	198266833	T	G	Het.			**nonsynonymous SNV**	17,3			970,35		1110		1	NM_012433: c.A2099C:p.K700T
73		exonic	ZMYM3	chrX	70461089	T	C	Het.			**nonsynonymous SNV**	42,7			694,38		178		1	NM_001171162: c.A3872G:p.Y1291C
74		exonic	POT1	chr7	124532367	T	C	Het.			**nonsynonymous SNV**	19,7			953,83		862		0,487	NM_015450: c.A77G:p.Y26C
75		UTR3	POT1	chr7	124462655	T	C	Het.	rs76436625	C=0.1611/807		45,7			5573,49		1272			
76		UTR3	POT1	chr7	124462661	C	T	Het.	rs17246404	T=0.1771/887			51,5			4781,48		906		
77		intronic	POT1	chr7	124477182	A	G	Het.	rs7787804	A=0.2983/1494		51,4	100		2631,89	5043,94	504	321		
78		exonic	HIST1H1E	chr6	26156822	C	A	Het.	rs148757707	T=0.0002/1	**synonymous SNV**	44,5			8453,91		1999			NM_005321: c.C204A:p.A68A
79		UTR3	NOTCH1	chr9	139389184	A	G	Het.	rs6563	A=0.4954/2481			45,6			1983,43		458		
80		exonic	NOTCH1	chr9	139391636	G	A	Het.	rs2229974	G=0.3049/1527	**synonymous SNV**	52,6	100		10985,4	27207,7	1998	1712		NM_017617: c.C6555T:p.D2185D
81		exonic	KIT	chr4	55602765	G	C	Het.	rs3733542	C=0.1641/822	**synonymous SNV**		51,4			2894,52		552		NM_000222: c.G2586C:p.L862L
82		intronic	TP53	chr17	7578115	T	C	Het.	rs1625895	T=0.1663/833		47,6	100		5865,63	11908,1	1253	744		
83		UTR5	TP53	chr17	7578645	C	T	Het.	rs2909430	C=0.1571/787		49,1	100		9869,35	31741,5	1998	1993		
84	**FTP:** first time point													3,352941176						
85	**LTP:** last time point																			
86	**Chr:** chromosome												median all	1						
87	**Ref.:** nucleotides in reference sequence																			
88	**Var.:** nucleotides in variant sequence																			
89	**MAF:** minor allele frequency																			
90	**SNV:** single nucleotide variation																			
91	**VAF: variant allele** frequency																			
92	**Qual:** estimated probability that the variant is due to chance (Phred quality score)																			
93	**Coverage:** number of times in which a given nucleotide was sequenced																			
94	**PP2 index:** PolyPhen2 prediction of functional effects of an amino acid substitution (PolyPhen2 range, 0 to 1)																			
95	**AA change:** localization of the alteration according to the format: Transcript (NM_00000): position and type of nucleotide variation: position and type of aminoacidic variation within the protein																			
