# Supplementary material for: Genetic dynamics in untreated CLL patients with either stable or progressive disease: a longitudinal study
Source: J Hematol Oncol. 2019 Nov 19;12:114. doi: 10.1186/s13045-019-0802-x (PMC6862808; doi:10.1186/s13045-019-0802-x)
Supplement: Supplementary file 5 — Additional file 5: Table S4. Characteristics of patients having few (dNV≤1) versus many (dNV>1) nucleotide variants. [file 13045_2019_802_MOESM5_ESM.docx]

| **S-CLL** | **IGVH-unmut** | **Zap70+** | **tri12** |
| --- | --- | --- | --- |
| **dNV>1** | 50,00% | 25,00% | 25,00% |
| **dNV≤1** | 0,00% | 14,29% | 0,00% |
| **p value** | 0.1091 | 1 | 0.3636 |
|  |  |  |  |
| **P-CLL** | **IGVH-unmut** | **Zap70+** | **tri12** |
| **dNV>1** | 55,56% | 44,44% | 33,33% |
| **dNV≤1** | 25,00% | 25,00% | 0,00% |
| **p value** | 0.3348 | 0.6199 | 0.2059 |
|  |  |  |  |
|  | **IGVH-unmut** | **Zap70+** | **tri12** |
| **dNV>1** | 53,85% | 38,46% | 30,77% |
| **dNV≤1** | 14,29% | 21,43% | 0,00% |
| **p value** | 0.0461 | 0.4197 | 0.0407 |

**Table S4:** Characteristics of patients having few (dNV≤1) versus many (dNV>1) nucleotide variants. Fisher’s exact test was used to test for significance.
